# Low-pass whole-genome sequencing reveals genomic diversity and ecotype-specific adaptation in indigenous Tigrayan chickens

**DOI:** 10.1038/s41598-026-54932-z

**Published:** 2026-09-02

**Authors:** Gebreslassie Gebru, Gurja Belay, Tsadkan Zegeye, Tadelle Dessie, Minister Birhanie, Mulalem Zenebe, Bashir Salim, Morris Katrina, Olivier Hanotte, Adriana Vallejo-Trujillo

**Affiliations:** 1 Tigray Agricultural Research Institute, P.O. Box 492, Mekelle, Tigray Ethiopia; 2 Department of Microbial Sciences and Genetics, College of Natural and Computational Sciences, Addis Ababa University, P.O. Box 1176, Addis Ababa, Ethiopia; 3 International Livestock Research Institute (ILRI), P.O. Box 5689, Addis Ababa, Ethiopia; 4 Camel Research Center, King Faisal University, 31982 Al-Ahsa, Saudi Arabia; 5 Centre for Tropical Livestock Genetics and Health (CTLGH), The Roslin Institute, University of Edinburgh, Easter Bush, Midlothian, EH25 9RG Scotland, UK; 6 School of Life Sciences, University of Nottingham, Nottingham, NG7 2RD UK

**Keywords:** Agroecology, Ecotype, Genetic diversity, Genetic structure, Inbreeding, Poultry, Computational biology and bioinformatics, Evolution, Genetics, Molecular biology

## Abstract

Indigenous chickens play a critical role in food security and climate resilience in smallholder systems, yet their genomic diversity and adaptive potential remain insufficiently characterised. This study employed low-pass whole-genome sequencing (LP-WGS; 0.2–1.99×) to investigate genomic diversity, population structure, inbreeding and candidate environment-associated genomic variation in 33 chickens from highland, midland, and lowland agroecologies in the Tigray region of northern Ethiopia. After imputation and stringent filtering, 23.4 million high-confidence SNPs were retained, including ~ 17% novel variants, indicating substantial uncharacterised genetic diversity in these populations. SNP density (13.8 ± 8.6 SNPs/kb) was comparable to values reported from high-coverage Ethiopian chicken datasets, demonstrating the suitability of LP-WGS for population genomics in resource-limited settings. Marked differences in genomic diversity were observed among ecotypes: midland chickens showed the highest nucleotide diversity (π = 0.00267), followed by lowland (π = 0.00233), whereas highland chickens showed the lowest diversity (π = 0.00203) and elevated genomic inbreeding (F_ROH_ and F_HOM_ ≈ 0.18). Population structure analyses revealed clear genetic separation among ecotypes. PCA (13.91% variation explained) distinguished lowland chickens along PC1 and separated highland from midland along PC2, while ADMIXTURE and *F*_*ST*_ patterns supported three major ancestral genomic backgrounds. Functional annotation of private missense variants uncovered distinct adaptive signatures reflecting the contrasting agroecological conditions. Highland chickens showed enrichment of candidate genes potentially involved in physiological processes relevant to high-altitude environments, including cold response, angiogenesis, cardiovascular regulation and metabolic homeostasis (eg., *PARP1*, *ACOX2*, *ITGB3*, *EDNRB*, *SOX8*, and *SOX10*). Midland chickens exhibited candidate signals of selection in genes with known roles in innate antiviral immunity, bacterial defence and inflammatory regulation (eg., *BAK1*, *CLSTN1*, *CYSLTR1*, *CYSLTR2*, *CXCR7*, *GIPR*, *DSCAM*, *GDAP1*, *TLR3*, *TLR4*, *TLR7*, *IFIH1*, *ADORA1*, *EPHB1*, and *TMPRSS2*). Lowland chickens displayed candidate variants associated with heat-stress response, DNA damage repair, oxidative balance and cardiovascular support under extreme temperatures (e.g., *MLH1*, *BDKRB1*, *GPR19*, *FLT1*, *CCL18*, *TGM2,* and *RAMP3*). Overall, the results indicate substantial genomic differentiation among ecotypes and suggest candidate environment-associated genetic divergence across Tigray’s diverse agroecological zones. These populations may represent important reservoirs of adaptive genetic variation for climate-resilient poultry breeding, warranting further functional validation and conservation-oriented management.

**Supplementary Information:**

The online version contains supplementary material available at https://doi.org/10.1038/s41598-026-54932-z.

## Introduction

Domestic chickens (*Gallus gallus domesticus*) have a long history in the Horn of Africa, with the Tigray Region of northern Ethiopia proposed as one of the earliest entry corridors for chickens into the continent. The oldest known chicken bone in Africa, dated back to approximately 2400–2800 years before present, has been found in the Tigray region^1^. Since their introduction, chickens have been exposed to and shaped by a wide range of agroecological conditions^2–4^. The elevation of Tigray spans from 500 to more than 4000 m.a.s.l.^5^, generating sharp gradients in temperature, humidity, vegetation, and pathogen pressure. These environmental contrasts have likely driven local adaptation and the formation of distinct chicken ecotypes.

Despite their historical, cultural, and economic importance, indigenous chickens in Tigray have experienced a dramatic decline in recent years. Prior to the outbreak of the 2020–2022 war, the region was home to an estimated 7 million chickens, of which 4.9 million (70.23%) were indigenous, 1.36 million (19.5%) exotic, and 0.72 million (10.27%) hybrids^6^. The conflict devastated the livestock sector with approximately 4.3 million chickens, 61% of the population, lost, and 83% of the poultry breeding facilities and 88% of veterinary service centres destroyed^7^. A parallel assessment reported that 75% of smallholder farmers lost around three-quarters of their livestock, with poultry experiencing the highest loss (87%)^8^. This unprecedented erosion of livestock resources places the remaining indigenous chickens at significant risk and highlights the urgent need for interventions to guide recovery, conservation, and sustainable utilisation efforts.

Genetic studies of indigenous Tigrayan chickens remain scarce. Earlier research primarily addressed morphology^9–13^ or ecological characterisation^4^. Country-wide genomic analyses by^14,15^, which included several Tigrayan populations (Gijet, Hadush Adi, Meseret, Metkilimat, and Mihquan), primarily focused on midland agroecology populations. The Tigray region, however, encompasses far greater ecological extremes from exceptionally hot lowlands at 626 m.a.s.l. to cold high-altitude environments exceeding 3347 m.a.s.l., with chicken populations that have adapted to distinct thermal, nutritional, and disease challenges across these environments.

Given the pronounced environmental heterogeneity of the region and the substantial erosion of indigenous chicken populations resulting from the rapid introduction of exotic commercial breeds and subsequent admixture, a comprehensive assessment of the genomic diversity, population structure, and adaptive mechanisms of Tigrayan indigenous chickens is urgently required. This study employs low-pass whole-genome sequencing to characterise genome-wide variation and identify ecotype-specific adaptive signatures across highland, midland, and lowland chickens. The findings aim to inform conservation strategies, support genetic improvement programmes, and contribute to the long-term resilience of indigenous poultry resources in northern Ethiopia.

## Materials and methods

### Description of sampling sites

**Fig. 1:**
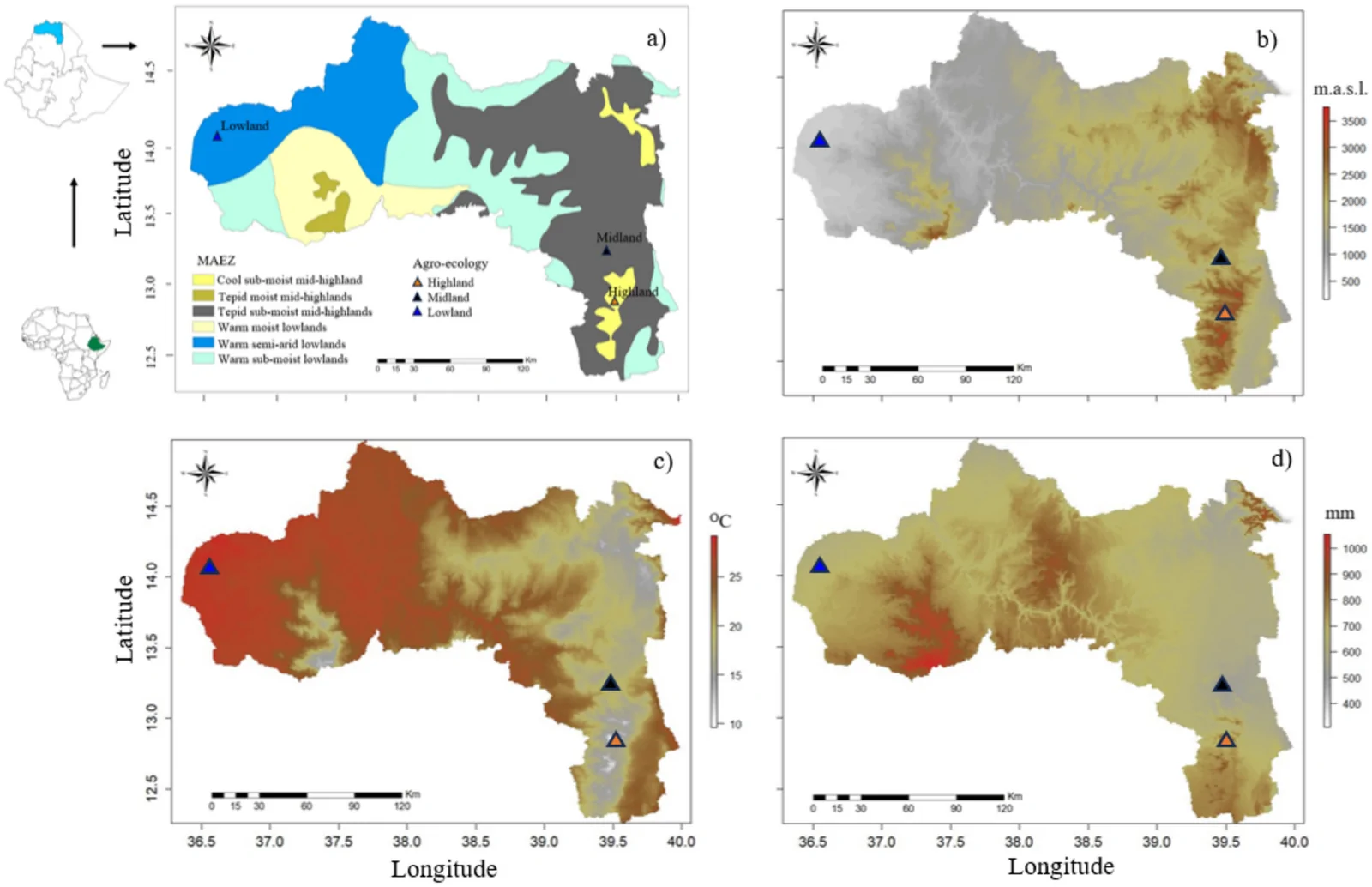
Blood sample collection sites. (**a**) Major Agro-ecological Zone (MAEZ), (**b**) elevation, (**c** annual temperature, and (**d**) annual precipitation.* Source*: (MOA, 2001; https://www.worldclim.org/).

The study was conducted in the northern part of Ethiopia, in the Tigray region, between 12°15′2'' and 14°57′2'' N latitude and 36°27′2'' and 39°59′2'' E longitude. According to traditional agroecological classification based on temperature and elevation, the region is categorised into highland (8%), midland (39%), and lowland (53%)^5^. Figure 1 illustrates the study region, including major agro-ecological zones, elevation, annual temperature, and annual precipitation. Detailed descriptions of the sampling villages are provided at Gebru et al.^16^. The chickens sampled in this study had not been previously investigated. The present study included chickens sampled from, Enda Mekoni (Tsibet), a cold highland environment at 3347 m.a.s.l. (referred to as highland); Hintalo Wajirat (Meseret), a mid-altitude environment at 2158 m.a.s.l. (referred to as midland); and Kafta Humera (May Kadra), a hot lowland environment 626 m.a.s.l. (referred to as lowland). These sites capture the major environmental gradients inhabited by indigenous Tigrayan chickens.

### Samples

Indigenous chickens were obtained from smallholder farmers in the study villages, with prior informed consent from each owner. Mature birds were selected, including males approximately one year of age and females around seven months of age, all of which had produced at least one clutch of chicks. Only blood samples were collected humanely from 33 indigenous chickens (14 males and 19 females) with 11 chickens from each of the three agroecologies (highland, midland, and lowland), following standard veterinary procedures without the use of anaesthetic agents.

The 33 samples analyzed in this study do not represent formally recognized breeds. Rather, they belong to indigenous, traditionally managed chicken populations, that had not been genetically characterized or classified into distinct breeds. Domestic chickens were likely introduced around 2800 years ago in the studied region^1^, after which they dispersed and adapted under local management practices to diverse environmental conditions. Accordingly, the chickens sampled in this study are best described as local populations originating from a shared ancestral background, but are currently distributed across three distinct agroecological zones in Tigray (highland, midland, and lowland).

After sampling, all chickens were returned to their owners and continued their normal daily activities without any intervention. Approximately 50–250 µl of blood was drawn from the wing vein using a 3 ml syringe and transferred to 2 ml cryovials containing 1.5 ml of absolute ethanol to preserve DNA. Samples were transported on ice and stored at − 20 °C until processing. All methods were carried out in accordance with relevant guidelines and regulations, including the ARRIVE guidelines^17^. Also, all animal handling and sampling procedures were approved by the Institutional Animal Care and Use Committee (IACUC) of the International Livestock Research Institute (ILRI-IREC2017-26).

### DNA extraction and sequencing

Genomic DNA was extracted from whole blood using the Qiagen DNeasy Blood and Tissue Kit, following the manufacturer’s protocol, at the molecular laboratory of the International Livestock Research Institute (ILRI) in Addis Ababa, Ethiopia. DNA integrity was assessed by agarose gel electrophoresis (Suppl. Figure S1), and the concentrations were measured using a Nanodrop spectrophotometer (Suppl. Table S1). All DNA samples exceeded 50 ng/µl and were subsequently submitted to SkimSeek™ (Neogene Europe Limited) for low-pass whole-genome sequencing (LP-WGS). Sequencing coverage ranged from 0.2× to 1.99× .

Genotype imputation was conducted using the Gencove low-pass sequencing pipeline (Gencove Inc., New York, USA) with the “Chicken low-pass v1.0” configuration, which uses the *Gallus gallus* reference genome (*GRCg6a*/*galGal6*). The imputation reference panel was constructed using high-coverage whole-genome sequencing data from the European Nucleotide Archive (ENA), including chicken populations from Ethiopia (PRJEB39275), Nigeria (PRJEB39536) and Tanzania (PRJEB39652). Ethiopian indigenous chicken (PRJEB39275) includes 245 individuals representing 25 Ethiopian chicken populations, including five Tigrayan midland populations (Gijet, Hadush Adi, Meseret, Metkilimat, and Mihquan). A comprehensive description of the population structure and sampling strategy for this dataset is provided in^14^, and the sequencing data (average genome coverage of about 57X) have been reported in detail in^18^. Nigerian indigenous chicken (PRJEB39536) comprises 120 individuals from 14 populations (average genome coverage of about 64X). Detailed information about these populations is described in^19^. Tanzanian indigenous chickens include 46 samples from 17 distinct populations (sample size of 1–7 per population, average genome coverage of about 44X). Raw sequencing reads were processed with GATK v4 to perform variant calling, yielding approximately 19.5 million genome-wide variants. The detected variants were subsequently phased to generate a high-quality haplotype reference panel for downstream genotype imputation analyses. Imputation was performed using the default parameters of the Gencove low-pass pipeline. Sex chromosome markers were excluded, and only autosomal SNPs were retained for downstream analyses.

### Variant filtration

A total of 33 samples were initially submitted for sequencing; however, one sample failed quality control at the sequencing facility due to insufficient sequencing quality and was therefore excluded from downstream analyses. Consequently, all genomic analyses in this study were conducted on 32 individuals. VCF files generated for 32 individuals were processed using BCFtools v1.11^20^. Filtering retained high-confidence autosomal SNPs that satisfied the following criteria: imputation quality scores (INFO or R^2^) ≥ 0.8, genotype call rate > 90%, biallelic SNPs only, exclusion of indels, and minor allele frequency (MAF) > 2%. Allele frequency metrics were added using BCFtools fill-tags plugin v1.11^20^, and SNP identities confirmed using dbSNP Galgal6 with the annotation option set to “known”.

### Reference populations

Notably, the 141 publicly available whole-genome sequences (WGS) of reference are distinct from the chickens sampled in the present study. This dataset was included solely as reference samples to assess the genetic diversity of Tigrayan chickens within a broader comparative framework. It comprises 30 non-Ethiopian domestic chicken genomes ((two West African, Ivory Coast and Cameroon), two Egyptian Fayoumi, five Saudi (Gallus_Saudi)), 21 wild junglefowl genomes (*Gallus gallus murghi*, *G. g. bankiva*, *G. g. spadiceus*, and *G. g. gallus* (accessions PRJNA552030 and PRJNA453469)), 63 non-Tigrayan Ethiopian indigenous chicken genomes highland (Alfamidir (n = 10) and Negasi Amba (n = 10)), midland (Jarso (n = 14) and Kumato (n = 10)), lowland (Gesses (n = 9) and Hugub (n = 10)), accession PRJEB46494), and five previously sequenced Tigrayan chicken populations from midland areas (Gijet, Hadush Adi, Meseret, Metkilimat, and Mihquan; n = 48). Raw FASTQ files were aligned to the *GRCg6a* (*Galgal6*) reference genome using the BWA-MEM, and variants were called following the GATK v3.8^21^ Best Practice, including HaplotypeCaller and Variant Quality Score Recalibration (VQSR). High-quality autosomal SNPs^18^ were retained for downstream analyses.

### Variant basic statistics, annotation and functional prediction

Genetic diversity parameters, including SNPs count, private SNPs, heterozygous and homozygous variants, transition-transversion ratio, and SNP density at 1 kb resolution, were calculated using VCFtools v0.1.15. SNP density across the genome was visualised using non-overlapping 10 kb windows. Nucleotide diversity (π) was calculated using 20 kb sliding windows with a 10 kb step size^22^. Observed heterozygosity (*H*_*O*_) was computed using the formula^23^:

$${H}_{o}=1-\frac{\text{Number of observed homozygotes (O(HOM))}}{\text{Number of non-missing autosomal SNPs (N\_Sites)}}$$

The shared vs. unique SNPs were illustrated using a Venn diagram generated in R v4.2.2^24^ using the ‘VennDiagram’ package^25^. Variant effects were annotated using Ensembl VEP v110^26^, and functional enrichment of overlapping missense variants was performed using DAVID v6.8, with significance set at *P* < 0.05 for identified GO terms and KEGG pathways^27^. This was done only for the 32 indigenous Tigrayan chicken analysed here.

### Run of homozygosity

Runs of homozygosity were detected using PLINK v1.9^28^, following parameters optimised for avian genomes. Specifically, an ROH was required to include a minimum of 50 SNPs, analysed within a sliding window of 50 SNPs, allowing up to five missing SNPs and no more than three heterozygous SNPs per window^23^. The minimum SNP density was set at 1 SNP per 50 kb, with a maximum gap density of 1 Mb between SNPs and a minimum ROH length of 300 kb. ROH were categorised as short (< 1 Mb), medium (1–8 Mb), and long (> 8 Mb)^29^.

### Genomic inbreeding coefficients

Two complementary inbreeding metrics were calculated. F_ROH_ was estimated as the proportion of the autosomal genome covered by ROH, using the formula F_ROH_ = L_ROH /_ Lauto, where Lauto = 1.1 Gb for the Galgal6 autosomal genome. F_HOM_ was calculated using PLINK’s “–het” function following^30^, using the formula F_HOM_ = (O–E) / (L–E), where O is the observed number of homozygotes, E the expected number under Hardy–Weinberg equilibrium, and L the total number of autosomal SNPs. The inbreeding coefficient was calculated in standard units (0–1). For presentation in the results section, we multiplied these values by 100 to express them as percentages. Figures display the raw inbreeding coefficient values (0–1) to maintain consistency with standard plotting conventions.

### Population structure analysis

Principal Component Analysis (PCA) was conducted using ~ 23 million SNPs in PLINK v1.9, and visualisation was performed in R v4.2.2 using the ggplot2 package. Admixture analysis was carried out using ADMIXTURE v1.3.0^31^, with K values ranging from 2 to 10. The optimal number of ancestral components was determined following the^32^ method, and plots were generated in R.

### Genomic differentiation

Pairwise genetic differentiation (*F*_ST_) among ecotypes and reference populations was computed using VCFtools v0.1.15 with 20 kb windows and 10 kb step sizes^22^. Heatmaps and dendrograms of weighted *F*_ST_ values were generated using the ggdendro, reshape2, and ggplot2 packages in R. A Neighbor-Net tree was constructed using SplitsTree5 v5.0.0 to visualise phylogenetic relationships^33^.

## Results

### SNP statistics

The Low-Pass Whole Genome sequencing (0.2–1.99X) from Tigrayan highland, midland, and lowland ecotypes generated a total of 39 million (M) raw SNPs (highland: 10.6 M, midland: 13.5 M, lowland: 15.2 M) (Suppl. Table S2). After applying the variant-filtrating criteria, 23.4 million high-quality autosomal SNPs were retained for downstream analyses (Suppl. Table S3). Among the three ecotypes, the midland population had the highest number of filtered SNPs (8.9 M), followed by the lowland (7.4 M) and highland (7.1 M) chickens. Consistently, the midland ecotype also showed the largest number of private SNPs (8.4 M), compared with the lowland (6.9 M) and highland (6.6 M) ecotypes.

Across all ecotypes, heterozygous SNPs were more abundant than homozygous SNPs (54–61% vs. 39–46%) (Suppl. Table S3 and Suppl. Figure S2). In total, indigenous Tigrayan chicken contributed 3.4 million novel SNPs, representing 17% of all detected variants, with the lowland ecotype showing the highest proportion of novel SNPs (17.38%), followed by the midland (16.97%) and highland (16.83%) populations (Suppl. Table S4). Novel SNP abundance decreased linearly with the chromosome size, with the largest macrochromosomes (1–7) carrying the highest number of novel SNPs, whereas chromosome 32 contained the fewest, consistent with its smaller size (Suppl. Figure S3).

Across the three ecotypes, heterozygous SNPs exceeded homozygous SNPs by 7.8% in highland, 21.1% in midland, and 22.6% in lowland chickens. The midland (2.3 M) and lowland (2.1 M) chickens had substantially more heterozygous SNPs than highland chickens (1.7 M). The midland ecotype showed the highest number of transitions (6.3 M) and transversions (2.6 M). In contrast, the highland (5.0 M Ts; 2.1 M Tv) and lowland (5.2 M Ts; 2.2 M Tv) ecotypes exhibited comparable substitution profiles (Suppl. Table S5). SNP sharing across ecotypes was low (0.58%), with the largest overlap observed between the midland and lowland (0.86%), and the smallest between the highland and midland ecotypes (0.80%) (Suppl. Figure S4).

**Fig. 2:**
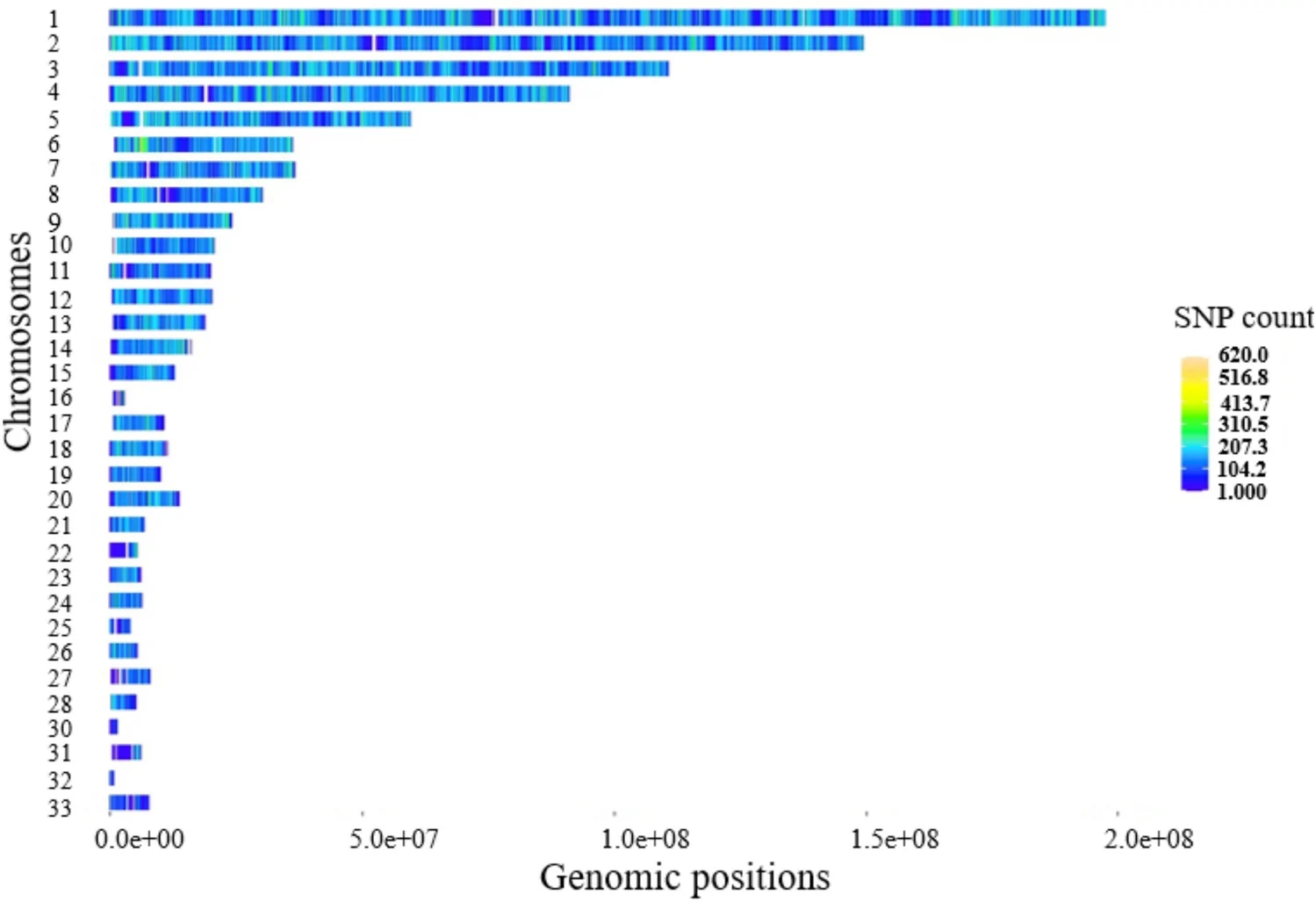
Chromosome-wise SNP distribution heatmap (non-overlapping 10 kb window): for the genome of Tigrayan indigenous chicken ecotypes.

The mean SNP density across the genome of Tigrayan chickens was 13.8 ± 8.6 SNPs per kilobase (kb), equivalent to approximately one SNP every 73 bp (Suppl. Figure S5). Chromosomes 6, 9, and 4 exhibited the highest SNP densities (15.8, 15.6, and 14.7 SNPs per kb, respectively), whereas chromosomes 31, 30, and 32 displayed the lowest (4.4, 4.4, and 5 SNPs per kb). A heatmap of SNP density across 10 kb windows (Fig. 2) showed that the majority of windows contained 102–184 SNPs, with these values occurring more than 500 times genome-wide, whereas high SNP counts (311–620 SNPs per window) were rare (< 10 times).

### Genetic similarity and dissimilarity of the indigenous chicken ecotypes

#### Observed heterozygosity and nucleotide diversity (π)

The observed heterozygosity (*Ho*) and nucleotide diversity (π) showed clear differences among the Tigrayan chicken ecotypes (Table 1). The lowland ecotype exhibited the highest *Ho* (0.29), followed by the midland ecotype (0.26), while the highland ecotype recorded the lowest heterozygosity (0.24) (Suppl. Figure S6). Within both the highland and midland populations, two individuals per ecotype showed markedly reduced genetic variability (< 0.20), whereas all lowland individuals displayed consistently higher variability (0.26–0.31). A similar pattern was observed for nucleotide diversity. The midland ecotype showed the highest π value (0.00267), followed by the lowland ecotype (0.00233), while the highland ecotype exhibited the lowest nucleotide diversity (0.00203) (Table 1). However, the π values of all three ecotypes were lower than those of external reference populations, including Ethiopian indigenous chickens (0.00323–0.00395), West African chickens (0.00368), Gallus_Saudi (Asian) (0.0032), and wild junglefowl (0.00355–0.0048). These comparisons suggest that Tigrayan chickens possess reduced genome-wide diversity compared to broader African, Asian, and wild lineages. The transition to transversion (Ts/Tv) ratio was highly consistent across the ecotypes, with values of 2.40, 2.41, and 2.39 in the highland, midland, and lowland chickens, respectively, suggesting similar mutational patterns and SNP quality across populations.

**Table 1:** Nucleotide diversity (π) for the Tigrayan indigenous chicken ecotypes and reference populations genome.

Populations	N	nSNP	Nucleotide diversity (π)	Ts/Tv
Highland	10	7,096,512	0.00203	2.40
Midland	11	8,932,227	0.00267	2.41
Lowland	8	7,363,048	0.00233	2.39
Alfamidir	10	10,539,558	0.00323	2.49
Gesses	10	10,337,743	0.00344	2.48
Gijet	9	11,046,899	0.00375	2.48
Hadush_Adi	9	10,213,712	0.00374	2.49
Hugub	10	10,034,153	0.00345	2.48
Jarso	14	11,155,696	0.00328	2.49
Meseret	10	11,419,999	0.00380	2.48
Metkilimat	10	11,295,871	0.00378	2.48
Mihquan	10	11,428,789	0.00387	2.48
Kumato	10	11,606,289	0.00395	2.48
Negasi Amba	10	10,535,898	0.00336	2.48
Gallus_Fayoumi	2	1,937,199	0.00238	2.385
Gallus_bankiva	2	2,553,621	0.00378	2.381
Gallus_gallus	9	3,640,880	0.00377	2.368
Gallus_spadiceus	5	4,373,556	0.00480	2.364
Gallus_murghi	3	2,806,743	0.00355	2.373
Gallus_Saudi	5	3,183,056	0.00320	2.38
West_Africa	2	2,393,561	0.00368	2.38

#### Run of homozygosity

**Fig. 3:**
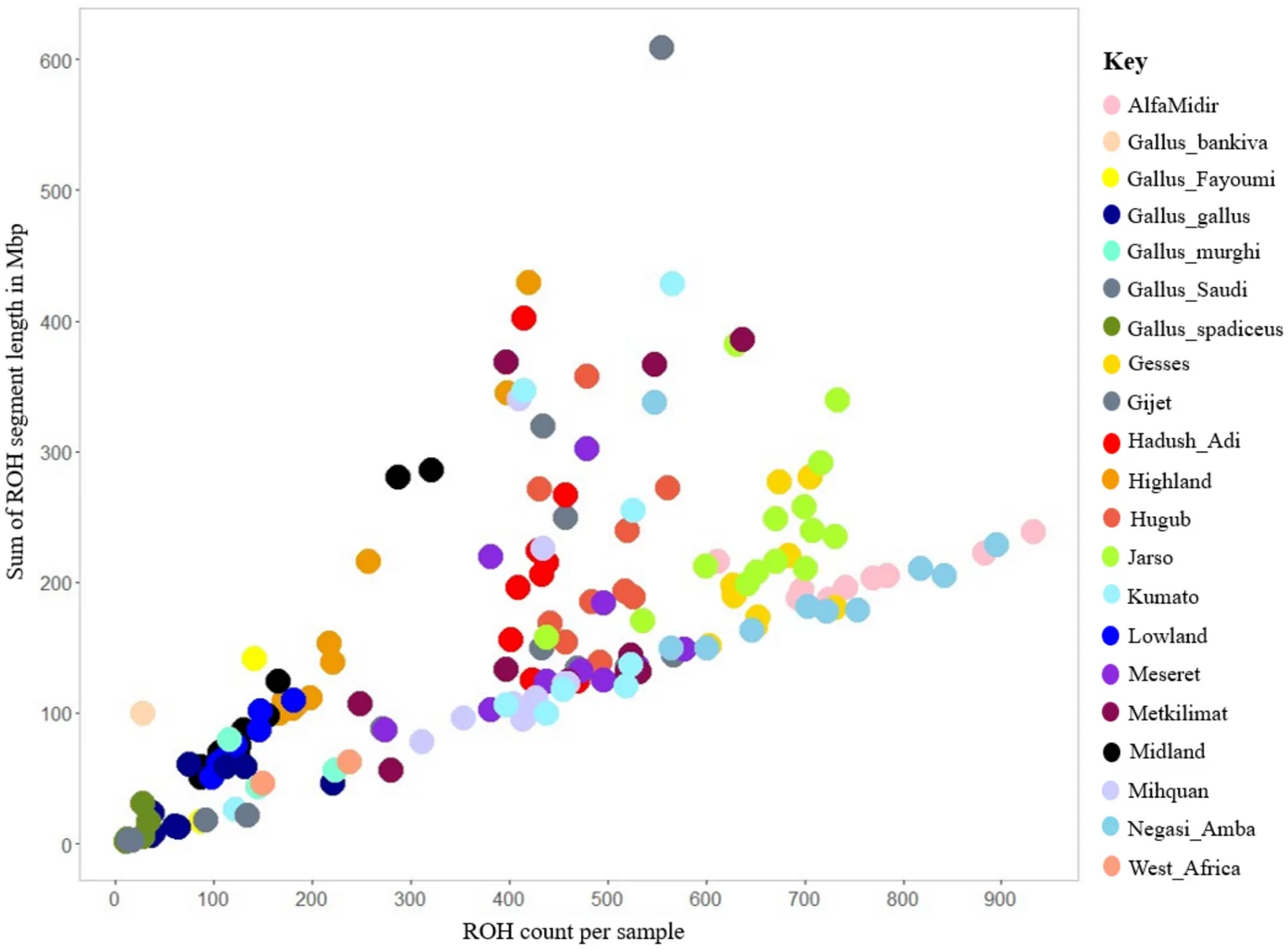
ROH count and length of the Tigrayan indigenous chicken ecotypes and reference populations.

The indigenous Tigrayan chicken ecotypes showed clear differences in the number, average length, and total length of ROH, as well as in the number of SNPs contained within ROH segments (Suppl. Tables S6 and S7, and Fig. 3). The highland ecotype exhibited the highest ROH burden, with 2,410 ROH segments, an average ROH length of 0.70 Mb, and a total ROH length of 1.80 Mb, whereas the lowland ecotype showed substantially fewer ROHs (1025), a shorter average length (0.59 Mb), and a much smaller total ROH span (0.62 Mb). The midland chickens displayed intermediate ROH characteristics, lying between the highland and lowland ecotypes.

The Tigrayan ecotypes had longer average ROH lengths (0.59–0.70 Mb) than the other populations. Ethiopian reference chickens ROH ranged from 0.27 to 0.50 Mb, while African and Gallus_Saudi (Asian) populations showed slightly higher values (0.29–0.60 Mb). Wild junglefowl showed even shorter ROH, ranging from 0.29 to 0.48 Mb. The number of ROHs detected in Tigrayan ecotypes (1025–2410) exceeded those of wild junglefowl, African, and Asian chicken (39–482), but remained lower than those reported for Ethiopian reference populations (3872–9121). The highland ecotype’s total ROH length (1.80 Mb) was similar to that observed in some Ethiopian populations (1.7–2.01 Mb), although it remained lower than that of the Hugub (2.2 Mb) and Jarso (3.4 Mb). In contrast, wild junglefowl, African, and Asian chickens had much shorter total ROH lengths (0.05–0.18 Mb). Across the three ecotypes, the number of SNPs within ROH regions ranged from 50 to 65,559 SNPs, reflecting substantial variation in ROH size and SNP content.

#### Genomic inbreeding coefficient

**Fig. 4:**
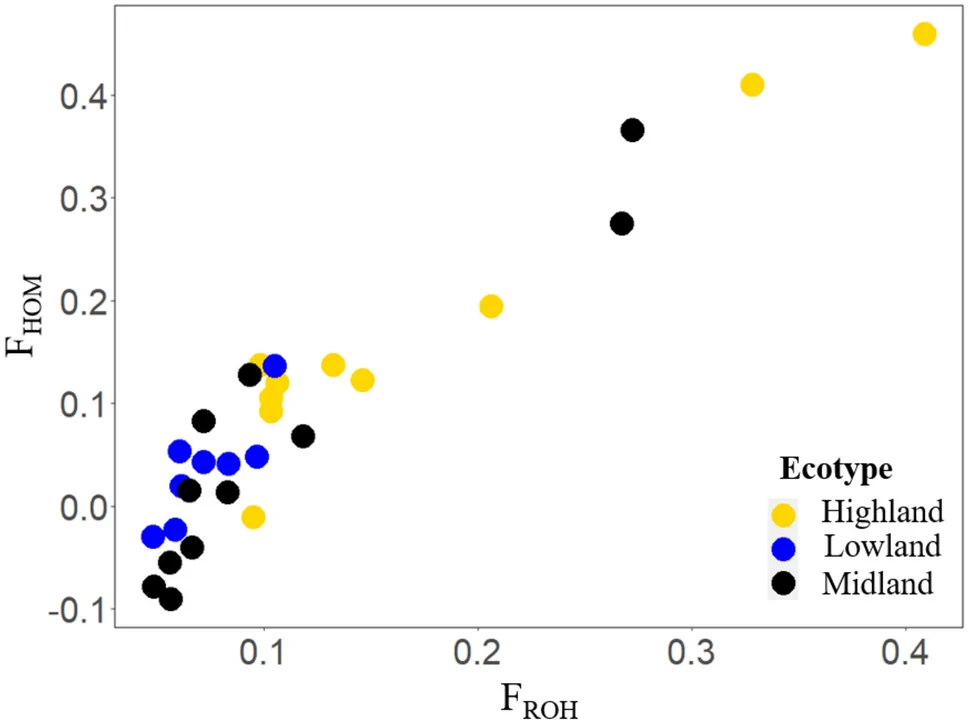
The inbreeding coefficients (F_ROH_ and F_HOM_) value of the Tigrayan indigenous chicken ecotypes.

The highland ecotype exhibited the highest inbreeding coefficient (18%), followed by the midland (6%) and lowland (4%) ecotypes (Fig. 4; Suppl. Figure S7). Nearly all highland chickens, except two, showed elevated F_HOM_ values (> 10%), indicating widespread excess homozygosity within this population. In contrast, all lowland individuals showed low F_HOM_ values (< 10%), except for a single chicken, and most midland individuals (eight of eleven) exhibited similarly low F_HOM_ values (< 10%). Genomic inbreeding, as measured by F_HOM_, was therefore most pronounced in the highland ecotype, suggesting reduced gene flow and greater relatedness within this group (Fig. 4). A smaller number of individuals from the midland (three) and lowland (one) ecotypes also showed evidence of genomic inbreeding (F_ROH_ > 10%), indicating that inbreeding is present but less common outside the highland environment.

### Population structure analysis

#### Principal component

**Fig. 5:**
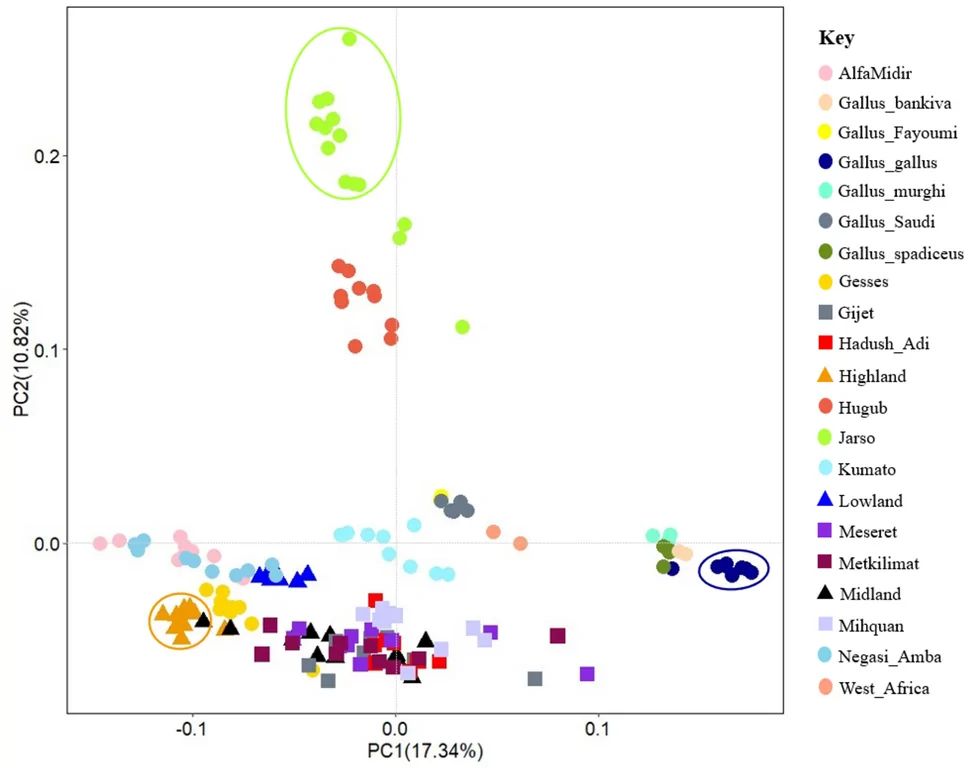
Principal component Analysis (PCA): PCA1 and PCA2 for the Tigrayan indigenous chicken ecotypes and the reference populations. (**a**), PCA1 vs PCA2 for the Tigrayan indigenous chicken Ecotypes and the Ethiopian reference chicken populations, (**b**), PCA1 vs PCA2 for the indigenous Tigrayan chicken ecotypes.

PCA revealed genetic structuring patterns among the indigenous Tigrayan chicken ecotypes and reference populations. Three clusters emerged: group 1 comprised the wild junglefowl populations, group 2 included the Ethiopian Jarso and Hugub populations, and group 3 contained the Tigrayan chicken ecotypes along with the remaining Ethiopian populations, Gallus_Fayoumi, Gallus_Saudi, and West_Africa chickens (Fig. 5). These latter two groups likely reflect the historical introduction routes and secondary dispersed waves of domestic chickens across Africa, separating ancestral lineages from more recent or regionally admixed populations. PCA1, which explained 17.34% of the total variation, differentiated Ethiopian indigenous populations from the wild junglefowl and the non-Ethiopian domestic populations (Gallus_Saudi, Gallus_Fayoumi, and West_Africa). PCA2 accounted for 10.82% of the variation and separated Jarso, Hugub, from all other chickens, suggesting a distinct demographic or introgression history for these two Ethiopian populations.

**Fig. 6:**
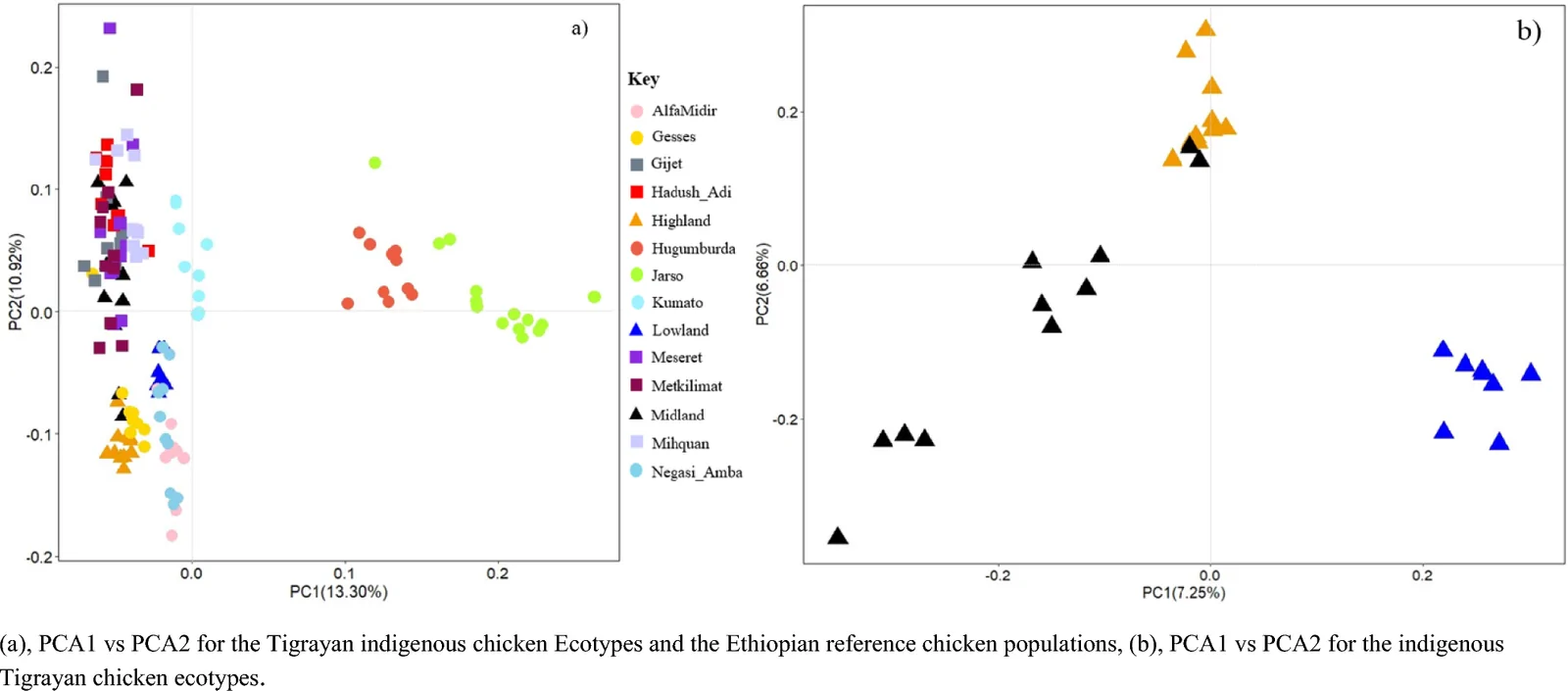
Principal component Analysis (PCA) for the Tigrayan indigenous chicken ecotypes and the reference population (Ethiopia).

A second PCA, focusing on Ethiopian chickens and Tigrayan ecotypes (Fig. 6a), captured 24.2% of the total variation (PC1 = 13.30%, PC2 = 10.92%). PC1 clearly separated Jarso and Hugub from the remaining populations, while PC2 distinguished the Tigrayan highland and lowland ecotypes, together with Alfamidir, Negasi_Amba, and Gesses, from the rest of the Ethiopian populations and midland ecotypes, indicating heterogeneous genetic backgrounds within Ethiopian chickens.

When the analysis was restricted to the indigenous Tigrayan ecotypes alone (Fig. 6b), PCA identified three genetically distinct clusters. PC1 explained 7.25% of the variation and separated the lowland ecotype from the highland and midland chickens. PC2 captured an additional 6.66% of the variation among highland, midland, and lowland Tigrayan ecotypes.

#### Admixture analysis

**Fig. 7:**
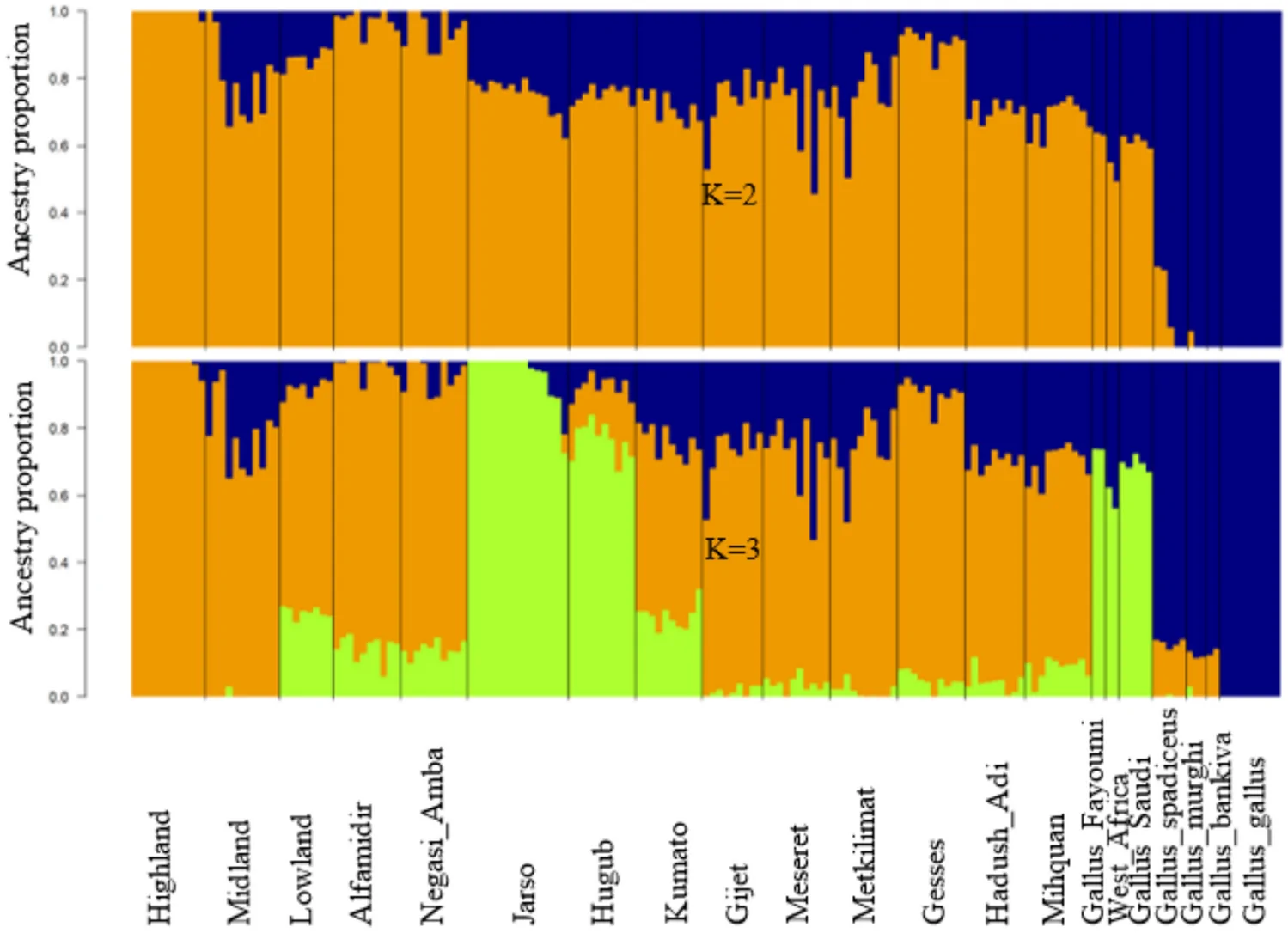
Admixture clustering diagram for the Tigrayan indigenous chicken ecotypes and reference populations.

The admixture plot at K = 3, identified as the optimal K, revealed three distinct ancestral genetic pools (Suppl. Figure S8). The admixture profiles of the Tigrayan indigenous chickens alongside those of the reference populations are presented in Fig. 7. At K = 2, two major genetic clusters were observed: one representing the shared ancestry with the wild junglefowl, and the other reflecting the dominant ancestry specific to the Tigrayan highland ecotype. At K = 3, the clustering pattern became consistent with the PCA results, resolving the three major genomic ancestries wild junglefowl, the Tigrayan highland ecotypes, and the Ethiopian Jarso and Hugub populations. These patterns indicate that multiple ancestral lineages have contributed differentially to the structure of Ethiopian indigenous chickens.

#### Genomic differentiation of indigenous Tigrayan chicken and the reference populations

The *F*_ST_ values highlighted varying degrees of genetic differentiation within and between the Tigrayan chicken ecotypes and reference populations (Suppl. Figure S9 and Suppl. Table S8). Among the three ecotypes, the highest genetic differentiation occurred between the highland and lowland populations (*F*_ST_ = 0.0336). The midland and lowland ecotypes showed slightly lower genetic differentiation (*F*_ST_ = 0.0271), whereas the lowest differentiation was observed between the highland and midland ecotypes (*F*_ST_ = 0.01233), indicating greater genomic similarity between these two ecotypes.

Comparison with other Ethiopian populations showed heterogeneous differentiation patterns. The highland ecotype exhibited high *F*_ST_ values with Jarso (*F*_ST_ = 0.1205), but showed significantly lower differentiation with Alfamidir (*F*_ST_ = 0.0252), Gesses (*F*_ST_ = 0.0271), and Negasi_Amba (*F*_ST_ = 0.0286). The lowland ecotype exhibited differentiation with Gesses (*F*_ST_ = 0.034985), and showed the highest differentiation with Jarso (*F*_ST_ = 0.1149) and Hugub (*F*_ST_ = 0.0958). The midland ecotype showed very low differentiation with Metkilimat (*F*_ST_ = 0.0019), Meseret (*F*_ST_ = 0.0033), and Gijet (*F*_ST_ = 0.0048), but much higher differentiation with Jarso (*F*_ST_ = 0.0948) and Hugub (*F*_ST_ = 0.0742).

**Fig. 8:**
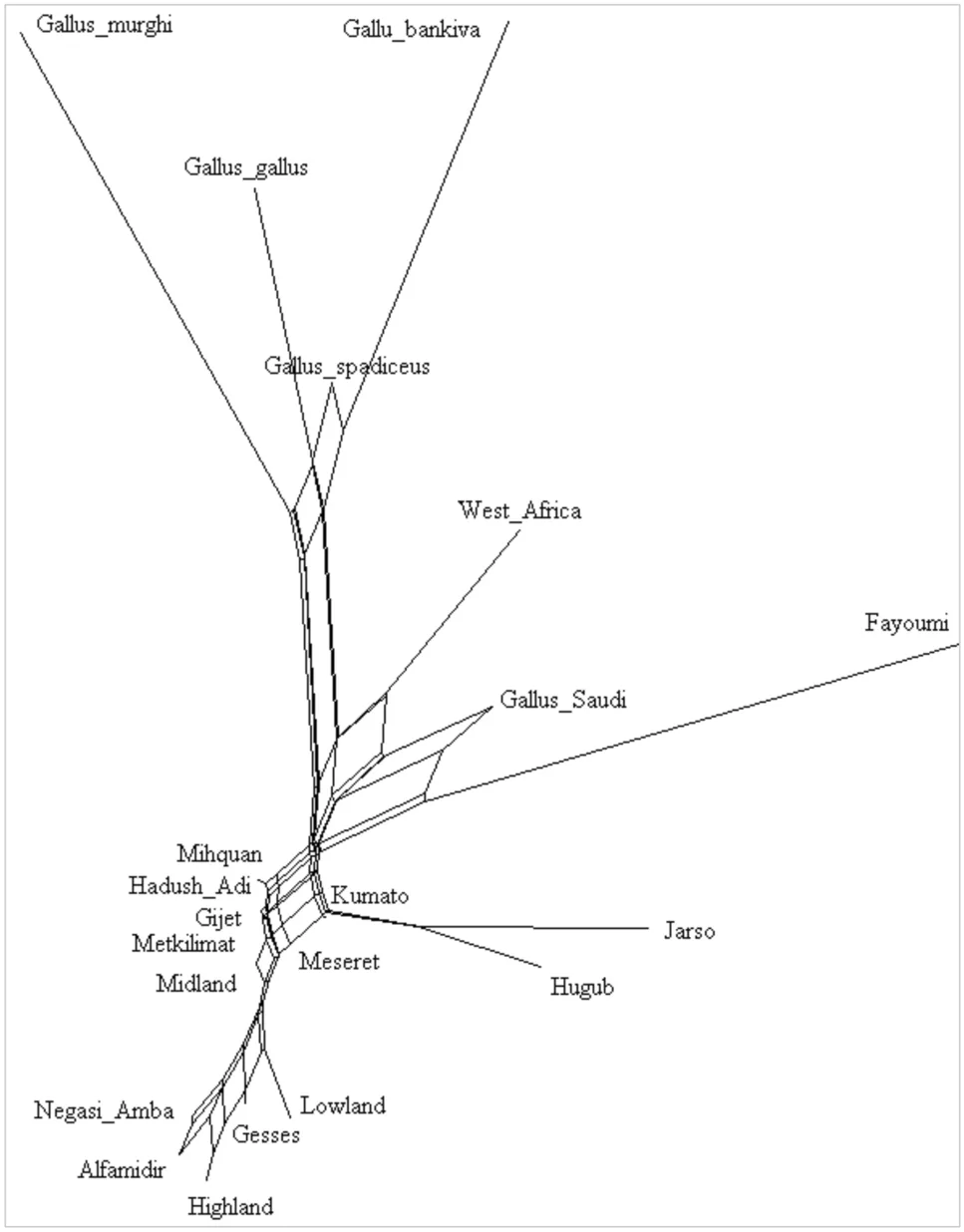
Neighbor-Net tree based on pairwise *F*_ST_ values between the Tigrayan indigenous chicken ecotypes and the reference populations.

The *F*_ST_ comparisons with wild junglefowl revealed substantial genomic divergence. The highland ecotype exhibited the highest *F*_ST_ values with wild ancestors, including *G. g. murghi* (*F*_ST_ = 0.2702), *G. bankiva* (*F*_ST_ = 0.2702), *G. gallus* (*F*_ST_ = 0.2322), and *G. spadiceus* (*F*_ST_ = 0.1976). Similar levels of divergence were observed for the midland and lowland ecotypes, with deep separation between Ethiopian indigenous chickens and wild ancestral lineages. The clustering patterns observed in the PCA (Fig. 5), admixture analysis (Fig. 7), and dendrogram (Suppl. Figure S9) were consistent with the genetic differentiation pattern revealed by the *F*_ST_. The Neighbour joining-Net tree constructed from pairwise *F*_ST_ values (Fig. 8) further supported the same population structure patterns, confirming the robustness of the observed genomic relationships.

#### Overlapping private SNPs annotation and functional enrichment analysis

From the VEP analysis of 23.4 million SNPs, intronic variants were the most abundant (55.9%), while intergenic, upstream-gene variants, and downstream gene variants accounted for 28.6–29%, 5%, and 6.7–6.9%, respectively, across the indigenous Tigrayan chickens (Suppl. Figure S10 and Suppl. Table S9). In the coding regions of the highland, midland, and lowland ecotypes, synonymous variants represented 71.2%, 70.7%, and 70.9%, respectively, while missense (non-synonymous) variants accounted for 28.4%, 28.9%, and 28.7% (Suppl. Figure S10d-f, and Suppl. Table S10).

For enrichment analysis, 790,698 private variants were identified in the highland ecotype, 1,413,192 in the midland, and 963,259 in the lowland (Suppl. Table S11). Within these, the highland, midland, and lowland ecotypes contained 1,995, 3,634, and 2,691 missense variants, respectively, along with 41, 69, and 41 missense splice-region variants. Variants predicted to have deleterious effects included 463, 808, and 608 missense variants, as well as 8, 15, and 12 missense splice-region variants in the highland, midland, and lowland ecotypes, respectively (Suppl. Table S12).

**Fig. 9:**
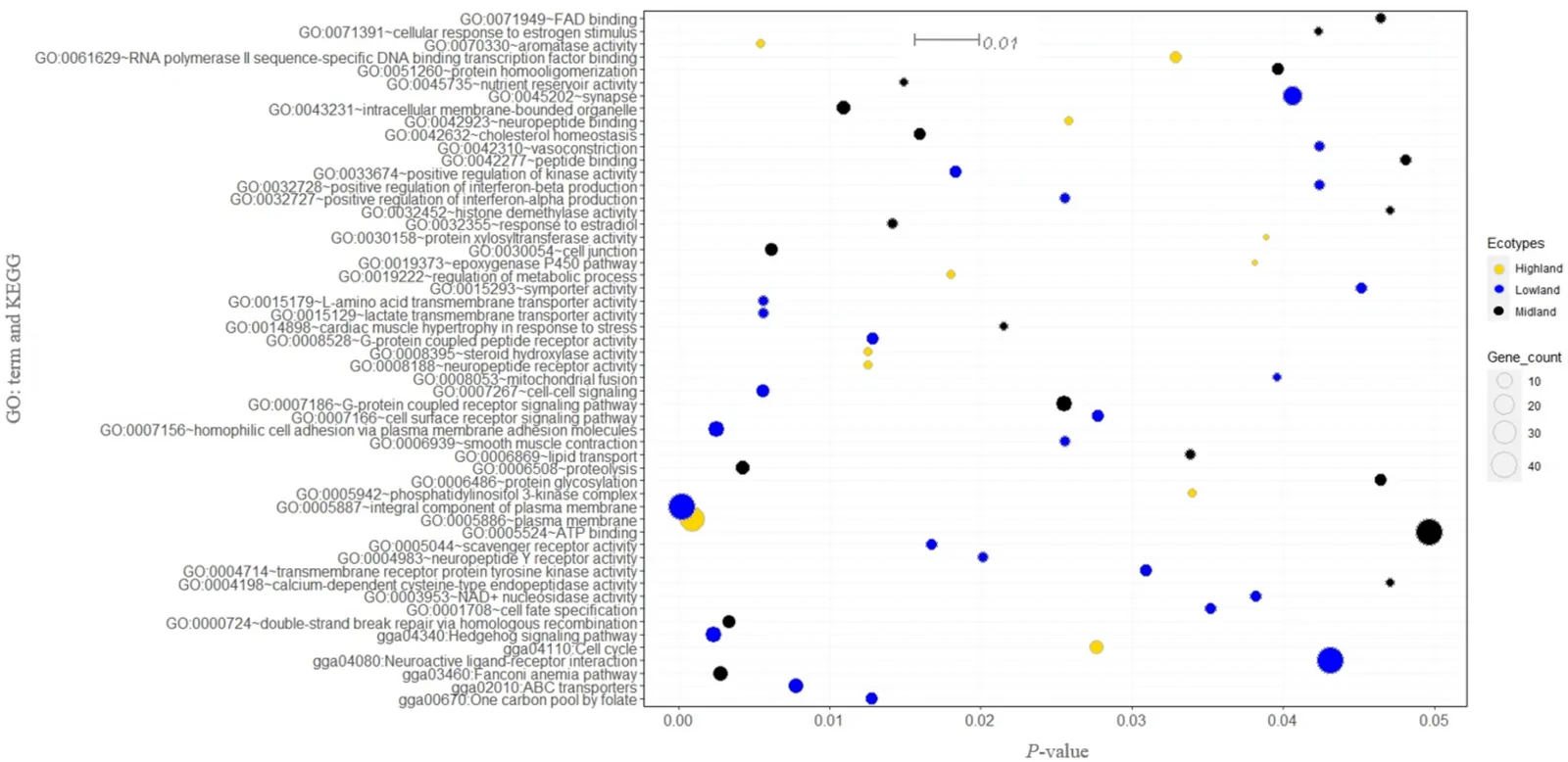
Private SNPs of the Tigrayan indigenous chicken ecotypes genome functionally enriched, Gene Ontology (GO): terms and Kyoto Encyclopedia of Genes and Genomes (KEGG) pathways.

The enrichment analysis (DAVID) of private missense variants revealed distinct functional categories and pathways among the ecotypes. There were 14, 29, and 35 significant GO terms (*P* < 0.0499) in the highland, midland, and lowland ecotypes, respectively, spanning Molecular Function (MF), Biological Process (BP), Cellular Component (CC), and KEGG pathways (Fig. 9). Four GO terms were shared across all three ecotypes: lipid transporter activity (GO:0005319), iron ion binding (GO:0005506), integral component of membrane (GO:0016021), and nuclear replication fork (GO:0043596), each involving multiple functionally diverse genes (Suppl. Table S13).

The highland and lowland ecotypes shared four GO terms: double-strand break repair via homologous recombination (GO:0000724), lipid transport (GO:0006869), nutrient reservoir activity (GO:0045735), and cellular response to estrogen stimulus (GO:0071391), in addition to one KEGG pathway: Fanconi anaemia (gga03460) (Suppl. Table S14). The midland and lowland ecotypes shared four GO terms: aspartic-type endopeptidase activity (GO:0004190), G-protein coupled receptor activity (GO:0004930), membrane (GO:0016020), and carbohydrate binding (GO:0030246), as well as two KEGG pathways: homologous recombination (gga03440) and cytokine-cytokine receptor interaction (gga04060) (Suppl. Table S15). No GO terms or KEGG pathways were shared exclusively between the highland and midland ecotypes, and missense splice-region variants did not yield any significant enrichments in the ecotypes.

Each ecotype possessed unique functional categories, suggesting ecotype-specific adaptations. The highland ecotype showed the fewest private GO terms, including axoneme (GO:0005930), DNA repair (GO:0006281), and enteric nervous system development (GO:0048484), as well as two KEGG pathways: peroxisome (gga04146) and ECM-receptor interaction (gga04512) (Suppl. Table S16 and Fig. 9).

The midland ecotype exhibited the highest number of GO terms (29) across BP, and MF and CC categories, including scavenger receptor activity (GO:0005044), neuropeptide signalling pathway (GO:0007218), transmembrane receptor protein tyrosine kinase activity (GO:0004714), integral component of plasma membrane (GO:0005887), G-protein coupled peptide receptor activity (GO:0008528), positive regulation of interferon-beta production (GO:0032728), homophilic cell adhesion via plasma membrane adhesion molecules (GO:0007156), cell–cell signalling (GO:0007267), cell surface receptor signalling pathway (GO:0007166), synapse (GO:0045202), and positive regulation of kinase activity (GO:0033674). Corresponding KEGG pathways include one-carbon pool by folate (gga00670), ABC transporters (gga02010), neuroactive ligand-receptor interaction (gga04080), and Hedgehog signalling pathway (gga04340) (Suppl. Table S17 and Fig. 9).

The lowland ecotype showed 14 significant GO terms, including calcium-dependent cysteine-type endopeptidase activity (GO:0004198), ATP binding (GO:0005524), protein glycosylation (GO:0006486), cardiac muscle hypertrophy in response to stress (GO:0014898), cell junction (GO:0030054), and intracellular membrane-bounded organelle (GO:0043231) (Suppl. Table S18 and Fig. 9).

## Discussion

Understanding the genomic architecture of indigenous livestock breeds is crucial for designing effective conservation strategies, improving productivity, and preparing production systems to withstand the mounting pressures of climate change. The genomic landscape of chickens in northern Ethiopia, particularly in the Tigray Region, reflects a long history of introductions, ecological exposure, and human selection. In this study, low-pass whole-genome sequencing (LP-WGS) was used to characterise genome-wide diversity, population structure, inbreeding patterns and ecotype-specific adaptive signatures among highland, midland and lowland Tigrayan chicken populations. LP-WGS is increasingly recognised as a cost-effective approach that can deliver high-density genomic data suitable for population genomics and functional inference, particularly in regions where high-coverage sequencing remains financially prohibitive^34–38^.

Although deep sequencing remains the gold standard^39^, previous studies have demonstrated that LP-WGS, combined with robust imputation panels, can approximate the accuracy of high-coverage datasets while dramatically reducing costs^40^. The present findings strengthen this evidence by showing that LP-WGS yielded 23.4 million high-confidence SNPs in indigenous chickens, comparable to variant numbers reported from conventional high-coverage sequencing of Ethiopian chickens^18,41^. The identification of approximately 17% novel SNPs further underscores how African poultry remain underrepresented in global chicken reference genomes and highlights the potential of Tigrayan chickens to contribute uniquely to the global chicken genomic landscape.

Across the three ecotypes, substantial variation was observed in genome-wide diversity indices. Midland and lowland chickens exhibited higher observed heterozygosity (*Ho*) and nucleotide diversity (π), reaching values comparable to wild Red Junglefowl and several genetically diverse indigenous Asian breeds^42,43^. Such high diversity suggests these populations have historically benefited from larger effective population sizes, broader gene flow and possibly lower levels of selective pressure associated with targeted breeding. In contrast, the lower diversity observed in the highland ecotype suggests a more constrained demographic history. This ecotype is found in colder, more geographically isolated regions characterised by steep landscapes, lower human population density, and reduced bird movement between households. These ecological and socio-economic conditions could restrict gene flow and promote genetic drift, contributing to the reduction in diversity. Similar patterns of reduced diversity have been reported in livestock subjected to ecological extremes or geographic isolation^44,45^. The highland environment of Tigray, characterised by hypoxia, cold temperatures, and pathogen-rich humid niches, likely imposed historical bottlenecks that shaped the observed genomic architecture.

The patterns of runs of homozygosity (ROH) provide further insight into the demographic forces shaping each ecotype. All three Tigrayan ecotypes carried ROH segments substantially longer than those found in many reference populations, a pattern consistent with spatial confinement, low levels of controlled breeding and limited genetic exchange among households^46^. However, the highland ecotype exhibited strikingly elevated ROH numbers, the longest total ROH lengths and higher genomic inbreeding coefficients (both F_ROH_ and F_HOM_). The magnitude of homozygosity suggests recent or ongoing inbreeding, potentially driven by restricted flock mobility and limited availability of breeding males in isolated, high-altitude communities. Long ROHs are typically indicative of recent shared ancestry^47^. Inbreeding has been associated with reducing productivity, fertility and disease resilience in poultry^48–50^. Conversely, midland and lowland chickens had lower genomic inbreeding and shorter ROHs, suggesting larger effective population sizes, more frequent exchange of breeding stock and greater genetic admixture. These ecotypes thus constitute important reservoirs of genetic diversity, offering opportunities for genetic improvement initiatives, if properly managed.

Population structure analyses reveal that genetic relationships among Tigrayan chickens reflect both ecological gradients and historical patterns of dispersal. PCA, ADMIXTURE and *F*_ST_ analyses consistently partitioned the chickens into three broad genomic backgrounds: wild junglefowl, the Ethiopian Jarso–Hugub lineage and a diverse domestic group containing all Tigrayan ecotypes. Within Tigray, the three ecotypes formed distinct clusters. Lowland chickens diverged strongly along the first principal component, reflecting adaptation to extreme heat and other low-altitude environmental pressures. Midland chickens occupied a distinct genomic space along the second principal component, reinforcing the notion that this ecotype represents a transitional but genetically cohesive group. The clustering patterns correspond with pairwise *F*_ST_ values, which showed the strongest differentiation between highland and lowland ecotypes and the lowest between highland and midland groups.

These genomic patterns are consistent with archaeological and historical accounts, which suggest multiple introduction waves of domestic chickens into Ethiopia (Suppl. Figure S11). Northern corridors through ancient Tigray and eastern routes via the Red Sea trade facilitated early chicken dispersal into the Horn of Africa^51–58^. Subsequent geographical partitioning, ecological pressures and locally adapted breeding practices likely contributed to the genomic divergence observed among ecotypes. The distinctiveness of Jarso and Hugub populations further supports a deep and complex history of domestic chickens within Ethiopia.

The functional annotation of private missense variants suggests potential ecotype-specific genetic differentiation with putative relevance to environmental adaptation. Several GO terms were shared across all ecotypes, including lipid transporter activity, iron ion binding, membrane components and DNA replication, reflecting fundamental physiological processes essential for survival in low-input scavenging systems^59–62^.

These shared processes suggest that, despite ecological differences, the chickens may retain core functionalities required for basic fitness in harsh production environments with limited feed and healthcare. Nevertheless, functional enrichments also revealed distinct adaptive signatures in each ecotype, strongly associated with their specific agroecologies. Highland chickens displayed enrichment of genes linked to cold response, cardiovascular regulation, angiogenesis, oxidative stress pathways and immune processes, which may be relevant to environmental conditions typical of high-altitude regions characterised by low temperatures and reduced oxygen availability. Genes such as *FRAS1*, *FREM2*, *LAMA3*, *ITGA9*, *ACOX2*, *EDNRB*, *SOX8* and *SOX10* contribute to feather development, skin integrity, blood vessel formation, metabolic regulation and detoxification^14,63–66^. These functional categories are consistent with reports of livestock adaptation to high-altitude conditions, which include enhanced vascularisation, improved thermogenic capacity, and increased stress resilience^67,68^. The functional profiles identified in highland chickens may reflect genomic regions with potential relevance to sensory processes, immune response and reproductive physiology under cold environmental conditions, where thermal stress and limited feed availability are common constraints.

Midland chickens exhibited the broadest functional enrichment patterns, which may be consistent with the ecological heterogeneity of mid-altitude agroecologies. Enrichment for pathways related to scavenger receptor activity, interferon-beta regulation, cell–cell signalling, ABC transporters and G-protein–coupled receptor activity suggests potential selection signals in genes involved in immune processes, detoxification and cellular communication^69–71^. Genes such as *TLR3, TLR4, TLR7, IFIH1, CYSLTR1, CYSLTR2, TMPRSS2* and *CXCR7* play key roles in antiviral signalling, bacterial recognition, inflammation control and immune cell trafficking^72–75^, and may contribute to ecotype-associated genomic differentiation.

The enrichment of angiogenesis-related genes (eg., *KDR* and *FLT1*) and development pathways (including Hedgehog signalling) may reflect genomic regions potentially relevant to physiological regulation in environmentally heterogeneous mid-altitude systems^76,77^. Genes associated with growth, muscle development, metabolic processes and reproductive function (e.g*., INSR, UTS2R, SHH, PCDH10, ADRA1A/B*) were also identified within differentiated regions, suggesting possible selection signals in loci linked to production and fitness-related traits under mixed crop–livestock systems^78–80^. Overall, the genomic patterns observed in the midland ecotype are consistent with ecotype-associated differentiation in pathways related to immune processes, metabolism and developmental regulation within a transitional agroecological context.

Lowland chickens exhibited pronounced genomic differentiation in regions containing genes previously implicated in heat response, DNA repair, metabolic regulation, cardiovascular function and immune processes. Enriched GO categories such as ATP binding, G-protein–coupled receptor signalling, cholesterol homeostasis and cardiac hypertrophy in response to stress may reflect pathways potentially relevant to physiological regulation under elevated temperatures^81–83^.

DNA repair genes, including *MLH1, MCM9, FANCA,* and *FANCD2*, were identified within differentiated regions and are known to participate in the detection and repair of DNA lesions, which could be relevant under chronic thermal stress conditions^84–86^. Immune-related genes such as *MUC6, TGM2, TRAP1* and *IFIH1* are involved in mucosal defence and antiviral responses^73,87,88^, and may contribute to ecotype-associated genomic divergence. The enrichment of loci such as *BDKRB1, CCL18, RAMP3* and *FLT1,* suggests possible selection signals in genes linked to oxidative stress response, inflammatory regulation and vascular function, which may be associated with high-temperature environments reported in the lowlands^89,90^. Additionally, genes related to metabolism, growth and reproduction (e.g., *VTG1–3, CAPN2, NUS1, FOXA1,* and *AOX1/2*)*,* were detected in differentiated regions, highlighting candidate pathways that could be relevant to physiological performance under heat-dominated production systems^91–93^.

It is important to note that the adaptive interpretations presented in this study are based on genomic variation, including population differentiation patterns and functional enrichment analyses. While these signals are consistent with putative local adaptation, they do not constitute direct evidence of functional or phenotypic effects. In the absence of gene expression, physiological, or experimental validation, the identified ecotype-associated variants and enriched biological pathways should be interpreted only as candidate genomic signatures potentially associated with environmental adaptation^94^. Future studies integrating transcriptomic, phenotypic, and experimental approaches will be essential to validate the functional roles of these candidate genome regions and genes.

In addition, the functional inference in this study is primarily based on variant annotation categories and GO/KEGG enrichment analyses. While these approaches provide biological context, they cannot by themselves distinguish neutral variation from variants shaped by natural selection, nor can they fully unravel demographic history from adaptive processes. The absence of explicit selection scan analyses (e.g., *F*_ST_ outlier tests or haplotype-based methods such as iHS or XP-EHH), therefore, represents a limitation of the present study. Consequently, the identified genes and pathways should be interpreted as putative candidates rather than definitive adaptive loci, and future studies integrating selection scans, genotype–environment association analyses, and functional validation will be required to confirm their adaptive significance.

Still, taken together, these results provide a unified genomic interpretation of differentiation and local adaptation among Tigrayan chicken ecotypes. Despite sharing a common ancestral origin and general scavenging production system, the ecotypes have diverged along ecological gradients that impose distinct thermal, nutritional, immunological and demographic pressures. Highland chickens display enrichment of genes potentially relevant to cold response, vascular adaptation and metabolic stability; midland chickens showed broad functional enrichment in immune and physiological pathways; and lowland chickens displayed candidate loci associated with heat response and DNA repair. These findings highlight putative genomic differences that could inform conservation, breeding and climate-resilient livestock improvement programs in northern Ethiopia and beyond.

The reduced genetic diversity and increased inbreeding observed in the highland ecotype suggest a potential susceptibility to genetic erosion. Conservation programs targeting the highland Tigrayan chicken should prioritise controlled mating, genetic rescue strategies and the introduction of new genetic material from genetically compatible populations. By contrast, the midland and lowland ecotypes, with higher genetic diversity and candidate adaptive alleles related to immunity and heat tolerance, could serve as valuable genetic resources for breeding programs aimed at enhancing resilience to environmental challenges and productivity improvement. With climate change increasing the frequency of heatwaves, disease outbreaks, and resource scarcity in sub-Saharan Africa, these ecotypes represent important reservoirs of genetic variation for sustainable poultry development.

Despite some limitations associated with sequencing depth (in this study 0.2× to 1.99×), which may influence genotype calling accuracy for low-frequency variants and genotype imputations relying on a reference panel that did not include all the Tigrayan ecotypes, LP-WGS emerges from this work as a practical and scalable approach for generating genome-wide information in resource-limited settings. It enables the identification of both diversity patterns and adaptive variants, thereby providing actionable insights for conservation and improvement of indigenous livestock. The genomic insights obtained here illustrate the biological richness of Tigrayan chickens and highlight the potential of new genomic tools to safeguard and utilise indigenous animal genetic resources that have sustained rural communities for centuries.

## Conclusion

This study reveals substantial genomic differentiation among highland, midland, and lowland Tigrayan chicken ecotypes, highlighting the strong influence of environmental, demographic, and management factors on their genetic structure. Low-pass whole-genome sequencing proved effective in capturing genome-wide diversity, detecting novel variants and identifying ecotype-specific adaptive signatures. Highland chickens showed reduced diversity and elevated inbreeding, suggesting potential vulnerability and the need for targeted conservation. In contrast, midland and lowland chickens exhibited higher diversity and enrichment of genes potentially involved in immune processes and heat-response pathways. The adaptive pathways uncovered here are consistent with the contrasting agroecological conditions of northern Ethiopia. These findings provide a genomic foundation for conservation planning, sustainable utilisation and climate-resilient breeding programmes aimed at improving poultry productivity and resilience in resource-limited settings. Future studies integrating larger sample sizes, higher and more uniform sequencing depth, population-specific reference panels, and individual-level environmental and phenotypic data will be important to strengthen the inferences from this study.

## Supplementary Information

## Data Availability

The raw sequencing data supporting the conclusions of this article are available at the NCBI Sequence Read Archive (SRA) under BioProject accession number [PRJNA1377557] (https://www.ncbi.nlm.nih.gov/bioproject/PRJNA1377557) .
