# Supplementary material for: Low-pass whole-genome sequencing reveals genomic diversity and ecotype-specific adaptation in indigenous Tigrayan chickens
Source: Sci Rep. 2026 Sep 2;16:27552. doi: 10.1038/s41598-026-54932-z (PMC13538659; doi:10.1038/s41598-026-54932-z)
Supplement: Supplementary file 1 [file 41598_2026_54932_MOESM1_ESM.docx]

Supplementary Figures and Tables: Low-pass whole-genome sequencing reveals genomic diversity and ecotype-specific adaptation in indigenous Tigrayan chickens

EH = highland; EL = lowland; EM = midland.

Suppl. Figure S1: DNA gel result of the sampled indigenous Tigrayan chicken ecotypes.

Suppl. Figure S2. Homozygous and heterozygous SNPs count across the indigenous Tigrayan chicken ecotypes genome.

Suppl. Figure S3. Chromosome-wise count of novel SNPs in the indigenous Tigrayan chicken genome.

Suppl. Figure S4. SNPs Venn diagram for the indigenous Tigrayan chicken ecotypes.

Chromosomes

Average SNP density per kb

Suppl. Figure S5. Chromosome-wise SNP density

Mean SNP density (count/kilobase-pair) per chromosome for the Tigrayan indigenous chicken ecotypes.

Suppl. Figure S6. Observed heterozygosity (individual and population average) value for the indigenous chicken ecotypes of Tigray, Ethiopia.

 Suppl. Figure S7. Inbreeding coefficient (individual and population average) value of the Tigrayan indigenous chicken ecotypes.

Suppl. Figure S8. The optimal number of genetic clusters is inferred by minimising the admixture cross-validation error using a cross-validation procedure.

 Suppl. Figure S9. Dendrogram and heatmap based on weighted *F*_ST_ (genetic distance) for the Tigrayan indigenous chicken ecotypes and the reference populations.

All consequences vs coding sequence, Highland, a and d; Midland, b and e; Lowland, c and f.

Suppl. Figure S10. Variant Effect Predictor (VEP) based sorting tolerant from intolerant (SIFT) analysis for the percentage of amino acid altering SNPs.

Suppl. Figure S11. Ancient trade routes to Ethiopia. Source: (<https://www.worldclim.org/>).

The Africa and Ethiopia maps were created by the authors using R [24] (version 4.5.1) with spatial packages including dplyr, ggplot2, raster, sp, sf, and tmap. Climate data (elevation) were obtained from the WorldClim database [95] (Version 2; <https://www.worldclim.org/>).

Suppl. Table S1. DNA concentration of the sampled Tigrayan indigenous chicken ecotypes.

| Sample Name | Ecotypes | Sex | Concentration ng/uL |
| --- | --- | --- | --- |
| EH4-03 | Highland | M | 57.42 |
| EH4-04 | Highland | F | 68.971 |
| EH4-05 | Highland | F | 67.452 |
| EH4-06 | Highland | M | 119.462 |
| EH4-07 | Highland | F | 86.697 |
| EH4-09 | Highland | F | 65.428 |
| EH4-10 | Highland | F | 52.622 |
| EH4-11 | Highland | F | 82.08 |
| EH4-12 | Highland | F | 83.686 |
| EH4-13 | Highland | M | 184.116 |
| EH4-14 | Highland | M | 65.383 |
| EH4-15 | Highland | F | 76.665 |
| EL7-01 | Lowland | F | 100.938 |
| EL7-02 | Lowland | F | 103.224 |
| EL7-03 | Lowland | F | 92.811 |
| EL7-04 | Lowland | F | 91.891 |
| EL7-05 | Lowland | F | 67.239 |
| EL7-07 | Lowland | M | 70.583 |
| EL7-08 | Lowland | M | 73.283 |
| EL7-10 | Lowland | M | 87.401 |
| EL7-11 | Lowland | M | 64.178 |
| EL7-12 | Lowland | M | 92.079 |
| EM4-01 | Midland | M | 174.024 |
| EM4-02 | Midland | M | 90.473 |
| EM4-03 | Midland | M | 100.196 |
| EM4-04 | Midland | M | 93.686 |
| EM4-05 | Midland | F | 136.012 |
| EM4-07 | Midland | F | 106.585 |
| EM4-08 | Midland | F | 74.338 |
| EM4-09 | Midland | F | 111.888 |
| EM4-10 | Midland | F | 55.651 |
| EM4-11 | Midland | F | 73.406 |
| EM4-12 | Midland | M | 131.013 |

EH = Highland; EL = Lowland; EM = Midland; ng = nanogram; uL = microliter; F = female; M = male.

Suppl. Table S2. Raw sequence information of the Tigrayan indigenous chicken ecotypes.

| **Ecotype** | **Sample ID** | **Number of SNPs** | **Bases Deduped Mapped** | **Effective Coverage** |
| --- | --- | --- | --- | --- |
| Highland | EH4-03 | 17,888,720 | 1,511,246,174 | 1.106299084 |
| Highland | EH4-04 | 6,595,135 | 393,381,875 | 0.28332461 |
| Highland | EH4-05 | 8,587,900 | 490,541,526 | 0.387538275 |
| Highland | EH4-06 | 15,976,841 | 1,292,200,189 | 0.910545994 |
| Highland | EH4-07 | 17,539,527 | 1,516,294,536 | 1.067566509 |
| Highland | EH4-09 | 6,567,966 | 397,159,870 | 0.281976218 |
| Highland | EH4-10 | 14,329,770 | 1,105,008,931 | 0.768039648 |
| Highland | EH4-11 | 7,255,653 | 370,087,173 | 0.316678197 |
| Highland | EH4-12 | 5,439,496 | 328,184,853 | 0.227518334 |
| Highland | EH4-13 | 5,260,323 | 286,582,972 | 0.219138207 |
| Highland | EH4-14 | 11,186,983 | 793,574,374 | 0.542155216 |
| Lowland | EL7-01 | 14,656,094 | 1,161,332,497 | 0.794706468 |
| Lowland | EL7-02 | 17,310,928 | 1,374,755,001 | 1.042999289 |
| Lowland | EL7-03 | 5,327,325 | 339,809,539 | 0.222263753 |
| Lowland | EL7-05 | 18,668,805 | 1,734,426,878 | 1.198660872 |
| Lowland | EL7-06 | 17,827,862 | 1,571,474,855 | 1.099439844 |
| Lowland | EL7-07 | 16,958,455 | 1,390,486,281 | 1.006264091 |
| Lowland | EL7-08 | 12,377,883 | 711,749,071 | 0.621865294 |
| Lowland | EL7-09 | 19,363,179 | 1,760,854,101 | 1.288727794 |
| Lowland | EL7-10 | 21,399,534 | 2,177,578,226 | 1.612237186 |
| Lowland | EL7-11 | 7,886,708 | 493,795,693 | 0.349618002 |
| Midland | EM4-01 | 17,958,084 | 1,483,392,257 | 1.114174833 |
| Midland | EM4-02 | 7,097,843 | 425,019,201 | 0.308607677 |
| Midland | EM4-03 | 13,951,177 | 1,018,872,441 | 0.737967082 |
| Midland | EM4-04 | 23,100,835 | 2,865,604,922 | 1.996608451 |
| Midland | EM4-05 | 15,783,521 | 1,211,738,563 | 0.892728924 |
| Midland | EM4-07 | 10,198,340 | 658,214,337 | 0.480492191 |
| Midland | EM4-08 | 13,448,729 | 920,257,224 | 0.699403764 |
| Midland | EM4-09 | 4,647,265 | 286,235,678 | 0.190984646 |
| Midland | EM4-10 | 11,817,567 | 675,586,818 | 0.583569387 |
| Midland | EM4-11 | 17,023,887 | 1,417,438,961 | 1.01298228 |
| Midland | EM4-12 | 13,872,991 | 1,052,849,932 | 0.731867671 |
| Highland average | | 10,602,574 | 771,296,588 | 0.56 |
| Midland average | | 13,536,385 | 1,092,291,849 | 0.80 |
| Lowland average | | 15,177,677 | 1,271,626,214 | 0.92 |

Suppl. Table S3. SNP statistics for the Tigrayan indigenous chicken ecotypes (individual and ecotype).

| Ecotypes | Population ID | Private SNPs | HH | HR | Total SNP |
| --- | --- | --- | --- | --- | --- |
| Highland | EH4-03 | 230,913 | 1,464,533 | 1,639,759 | 3,104,292 |
| Highland | EH4-04 | 171,803 | 1,669,956 | 1,199,700 | 2,869,656 |
| Highland | EH4-05 | 181,093 | 1,391,354 | 1,756,839 | 3,148,193 |
| Highland | EH4-07 | 220,706 | 1,400,146 | 1,786,977 | 3,187,123 |
| Highland | EH4-09 | 190,396 | 1,359,966 | 1,791,365 | 3,151,331 |
| Highland | EH4-10 | 288,214 | 1,277,633 | 2,057,452 | 3,335,085 |
| Highland | EH4-11 | 199,840 | 1,348,014 | 1,821,493 | 3,169,507 |
| Highland | EH4-12 | 217,706 | 1,339,433 | 1,848,021 | 3,187,454 |
| Highland | EH4-13 | 190,506 | 1,372,192 | 1,755,683 | 3,127,875 |
| Highland | EH4-14 | 149,756 | 1,722,532 | 1,099,147 | 2,821,679 |
| Midland | EM4-01 | 188,230 | 1,528,713 | 2,319,201 | 3,847,914 |
| Midland | EM4-02 | 132,353 | 1,858,298 | 1,578,340 | 3,436,638 |
| Midland | EM4-03 | 132,529 | 1,574,164 | 2,171,125 | 3,745,289 |
| Midland | EM4-04 | 276,408 | 1,380,275 | 2,714,643 | 4,094,918 |
| Midland | EM4-05 | 195,371 | 1,466,810 | 2,454,551 | 3,921,361 |
| Midland | EM4-07 | 214,204 | 1,378,086 | 2,625,941 | 4,004,027 |
| Midland | EM4-08 | 225,664 | 1,425,462 | 2,588,451 | 4,013,913 |
| Midland | EM4-09 | 175,489 | 1,513,003 | 2,282,239 | 3,795,242 |
| Midland | EM4-10 | 240,733 | 1,364,299 | 2,683,818 | 4,048,117 |
| Midland | EM4-11 | 196,247 | 1,461,766 | 2,450,518 | 3,912,284 |
| Midland | EM4-12 | 172,926 | 1,777,203 | 1,804,181 | 3,581,384 |
| Lowland | EL7-01 | 284,740 | 1,288,526 | 2,268,483 | 3,557,009 |
| Lowland | EL7-02 | 276,537 | 1,327,214 | 2,160,189 | 3,487,403 |
| Lowland | EL7-03 | 267,979 | 1,337,743 | 2,084,617 | 3,422,360 |
| Lowland | EL7-05 | 261,875 | 1,352,973 | 2,112,839 | 3,465,812 |
| Lowland | EL7-06 | 271,946 | 1,278,029 | 2,253,535 | 3,531,564 |
| Lowland | EL7-07 | 271,862 | 1,360,376 | 2,097,466 | 3,457,842 |
| Lowland | EL7-08 | 270,629 | 1,342,013 | 2,109,008 | 3,451,021 |
| Lowland | EL7-11 | 241,542 | 1,428,540 | 1,902,161 | 3,330,701 |
| Highland | | 6,596,061 | 1,434,576 | 1,675,644 | 7,096,512 |
| Midland | | 8,430,801 | 1,520,734 | 2,333,910 | 8,932,227 |
| Lowland | | 6,847,647 | 1,339,427 | 2,123,537 | 7,363,048 |
| Highland (HH and HR%) | |  | 53.9 | 46.1 |  |
| Midland (HH and HR%) | |  | 60.5 | 39.5 |  |
| Lowland (HH and HR%) | |  | 61.3 | 38.7 |  |

HH = homozygous; HR = heterozygous; EH = Highland; EM = Midland; WL = Lowland

Suppl. Table S4. Known and novel SNPs count for the Tigrayan indigenous chicken genome.

| Ecotypes | Known SNPs mm | Novel SNPs mm | Novel SNP% | Total SNPs mm |
| --- | --- | --- | --- | --- |
| Highland | 6.1 | 1.0 | 16.83 | 7.1 |
| Midland | 7.6 | 1.3 | 16.97 | 8.9 |
| Lowland | 6.3 | 1.1 | 17.38 | 7.4 |
| Total | 2.0 | 3.41 | 17.06 | 23.4 |

mm = million; % = percent.

Suppl. Table S5. Nucleotide transitions and transversions in the genome of Tigrayan indigenous chicken ecotypes.

| Ecotype | Sample ID | A>C | A>G | T>G | T>A | T>C | C>T | C>A | A>T | G>T | C>G | G>C | G>A | Ts | Tv |
| --- | --- | --- | --- | --- | --- | --- | --- | --- | --- | --- | --- | --- | --- | --- | --- |
| Highland | EH4-03 | 107779 | 526664 | 107124 | 124462 | 526705 | 569849 | 116317 | 124876 | 116282 | 106858 | 106473 | 570903 | 2194121 | 910171 |
| Highland | EH4-04 | 100073 | 489068 | 99173 | 115526 | 488155 | 525044 | 106954 | 114882 | 107446 | 98504 | 98453 | 526378 | 2028645 | 841011 |
| Highland | EH4-05 | 108841 | 534495 | 108567 | 126095 | 578615 | 534116 | 117502 | 126357 | 117751 | 108029 | 108195 | 579630 | 2226856 | 921337 |
| Highland | EH4-07 | 111258 | 540627 | 109884 | 128382 | 540337 | 585489 | 119149 | 128068 | 119998 | 109194 | 109072 | 585665 | 2252118 | 935005 |
| Highland | EH4-09 | 109604 | 535250 | 108566 | 126973 | 535678 | 577888 | 117648 | 126162 | 118327 | 108387 | 108074 | 578774 | 2227590 | 923741 |
| Highland | EH4-10 | 115580 | 564164 | 114710 | 134031 | 564629 | 613168 | 125175 | 133965 | 125553 | 114450 | 114322 | 615338 | 2357299 | 977786 |
| Highland | EH4-11 | 110304 | 537538 | 109249 | 127359 | 537603 | 581398 | 118661 | 126999 | 119092 | 109107 | 108594 | 583603 | 2240142 | 929365 |
| Highland | EH4-12 | 111084 | 541484 | 110021 | 128318 | 541175 | 584401 | 119214 | 128324 | 119561 | 110080 | 108865 | 584927 | 2251987 | 935467 |
| Highland | EH4-13 | 108647 | 531810 | 107825 | 125504 | 531741 | 572869 | 116793 | 125937 | 117324 | 107486 | 107118 | 574821 | 2211241 | 916634 |
| Highland | EH4-14 | 97855 | 481311 | 97514 | 113347 | 480250 | 516623 | 104739 | 113347 | 105453 | 96683 | 96693 | 517581 | 1995765 | 825631 |
| Midland | EM4-01 | 133109 | 654677 | 132443 | 153508 | 652724 | 708098 | 143187 | 152601 | 143523 | 132579 | 132400 | 709065 | 2724564 | 1123350 |
| Midland | EM4-02 | 119401 | 588720 | 118440 | 136737 | 587730 | 628733 | 126807 | 135878 | 127372 | 118383 | 117964 | 630473 | 2435656 | 1000982 |
| Midland | EM4-03 | 130212 | 639669 | 129006 | 149023 | 637703 | 687069 | 138949 | 149170 | 139143 | 128996 | 128461 | 687888 | 2652329 | 1092960 |
| Midland | EM4-04 | 141610 | 692686 | 140163 | 162497 | 692052 | 757327 | 152541 | 163002 | 153237 | 141056 | 140814 | 757933 | 2899998 | 1194920 |
| Midland | EM4-05 | 135435 | 666540 | 134960 | 155400 | 664220 | 722663 | 146094 | 155115 | 146587 | 134726 | 134813 | 724808 | 2778231 | 1143130 |
| Midland | EM4-07 | 138499 | 679759 | 137596 | 159353 | 678292 | 738426 | 148823 | 159170 | 149377 | 137974 | 137427 | 739331 | 2835808 | 1168219 |
| Midland | EM4-08 | 138718 | 680744 | 138027 | 159776 | 679254 | 740644 | 149377 | 160045 | 149885 | 138459 | 137860 | 741124 | 2841766 | 1172147 |
| Midland | EM4-09 | 132144 | 647082 | 130847 | 151468 | 645935 | 696049 | 140530 | 151225 | 141447 | 130672 | 129893 | 697950 | 2687016 | 1108226 |
| Midland | EM4-10 | 139814 | 686440 | 138657 | 160969 | 685368 | 747424 | 150746 | 160794 | 150903 | 139360 | 139189 | 748453 | 2867685 | 1180432 |
| Midland | EM4-11 | 135809 | 665956 | 134482 | 156132 | 663654 | 719823 | 145258 | 155867 | 146233 | 134258 | 134353 | 720459 | 2769892 | 1142392 |
| Midland | EM4-12 | 124472 | 611874 | 123605 | 142523 | 609810 | 657296 | 132381 | 142028 | 133088 | 123325 | 122395 | 658587 | 2537567 | 1043817 |
| Lowland | EL7-01 | 123257 | 602047 | 122901 | 143442 | 600775 | 655608 | 133300 | 142767 | 133585 | 121760 | 122127 | 655440 | 2513870 | 1043139 |
| Lowland | EL7-02 | 120894 | 591152 | 120926 | 140314 | 589720 | 641387 | 130514 | 140061 | 130678 | 119657 | 119876 | 642224 | 2464483 | 1022920 |
| Lowland | EL7-03 | 118855 | 580892 | 118951 | 138127 | 579296 | 629060 | 127878 | 137754 | 127914 | 117234 | 117490 | 628909 | 2418157 | 1004203 |
| Lowland | EL7-05 | 120240 | 586823 | 119889 | 139318 | 586271 | 637662 | 129574 | 139164 | 129862 | 119034 | 119248 | 638727 | 2449483 | 1016329 |
| Lowland | EL7-06 | 122386 | 597208 | 121647 | 142058 | 596206 | 650707 | 132349 | 141860 | 132636 | 121173 | 121599 | 651735 | 2495856 | 1035708 |
| Lowland | EL7-07 | 119860 | 584959 | 119580 | 139379 | 584664 | 637100 | 129813 | 138836 | 129817 | 118395 | 119009 | 636430 | 2443153 | 1014689 |
| Lowland | EL7-08 | 119309 | 585424 | 119417 | 138704 | 584249 | 635030 | 128815 | 138153 | 129627 | 118085 | 118669 | 635539 | 2440242 | 1010779 |
| Lowland | EL7-11 | 115961 | 565412 | 115543 | 133603 | 565130 | 611698 | 124455 | 133541 | 124920 | 114027 | 114301 | 612110 | 2354350 | 976351 |
| Highland average | | 236475 | 1133801 | 235050 | 285396 | 1133945 | 1368746 | 278096 | 285098 | 278350 | 245308 | 244351 | 1371896 | 5008388 | 2088124 |
| Midland average | | 295147 | 1415485 | 293541 | 355813 | 1412958 | 1740236 | 350732 | 354833 | 351725 | 309236 | 308568 | 1743953 | 6312632 | 2619595 |
| Lowland average | | 245703 | 1179093 | 245738 | 296729 | 1176472 | 1419220 | 287726 | 295476 | 288515 | 254054 | 254404 | 1419918 | 5194703 | 2168345 |

A = adenine; C = cytosine; G = guanine; T = thymine; Ts = transition; Tv = transversion; EH = Highland; EM =Midland; EL = Lowland.

Suppl. Table S6. ROH statistics in individual samples of the Tigrayan indigenous chicken ecotypes.

| Sample | Ecotype | No. of ROH | Total ROH length in kbp | Average ROH segment length kbp | Minimum ROH length kbp | Maxim ROH length kbp | SNPs |
| --- | --- | --- | --- | --- | --- | --- | --- |
|  |  |  |  |  |  |  |  |
| EH4-03 | Highland | 256 | 216608 | 846.125 | 300 | 7813 | 1440093 |
| EH4-04 | Highland | 398 | 344815 | 866.37 | 300 | 7250 | 2529904 |
| EH4-05 | Highland | 220 | 139158 | 632.534 | 300 | 3369 | 843120 |
| EH4-07 | Highland | 217 | 153585 | 707.763 | 302 | 4179 | 960688 |
| EH4-09 | Highland | 198 | 111424 | 562.749 | 301 | 2207 | 622427 |
| EH4-10 | Highland | 166 | 99718.8 | 600.715 | 300 | 6707 | 567543 |
| EH4-11 | Highland | 186 | 108452 | 583.073 | 301 | 3073 | 591655 |
| EH4-12 | Highland | 171 | 108648 | 635.371 | 302 | 5271 | 644819 |
| EH4-13 | Highland | 179 | 103281 | 576.988 | 301 | 3125 | 601532 |
| EH4-14 | Highland | 419 | 429291 | 1024.56 | 300 | 9468 | 3143972 |
| EM4-01 | Midland | 165 | 124350 | 753.634 | 301 | 3565 | 1063480 |
| EM4-02 | Midland | 321 | 285879 | 890.59 | 300 | 7486 | 2509207 |
| EM4-03 | Midland | 154 | 98229.7 | 637.855 | 304 | 3545 | 675205 |
| EM4-04 | Midland | 86 | 59234.6 | 688.774 | 300 | 6537 | 422888 |
| EM4-05 | Midland | 130 | 87287.3 | 671.441 | 303 | 3209 | 637076 |
| EM4-07 | Midland | 101 | 59005 | 584.208 | 300 | 2573 | 403974 |
| EM4-08 | Midland | 106 | 69591.7 | 656.525 | 300 | 4027 | 536441 |
| EM4-09 | Midland | 125 | 75432.5 | 603.46 | 301 | 2515 | 537192 |
| EM4-10 | Midland | 87 | 51126.8 | 587.664 | 301 | 3437 | 365361 |
| EM4-11 | Midland | 124 | 68510.3 | 552.503 | 300 | 2068 | 453391 |
| EM4-12 | Midland | 287 | 280885 | 978.692 | 301 | 6132 | 2529243 |
| EL7-01 | Lowland | 97 | 50750.8 | 523.204 | 304 | 1965 | 286881 |
| EL7-02 | Lowland | 110 | 64506 | 586.418 | 300 | 3011 | 350964 |
| EL7-03 | Lowland | 119 | 63578.1 | 534.27 | 303 | 1220 | 332970 |
| EL7-05 | Lowland | 146 | 87489.5 | 599.243 | 301 | 3415 | 525091 |
| EL7-06 | Lowland | 105 | 61644.2 | 587.087 | 303 | 4708 | 361329 |
| EL7-07 | Lowland | 147 | 101673 | 691.65 | 301 | 3477 | 621103 |
| EL7-08 | Lowland | 121 | 75381.6 | 622.988 | 302 | 2576 | 395097 |
| EL7-11 | Lowland | 180 | 110289 | 612.715 | 300 | 3388 | 687139 |

EH = highland; EM = midland; EL = lowland; No. Number: ROH = run of homozygosity; kbp = kilobase-pairs.

Suppl. Table S7. The number and length of ROH and SNP numbers in the ROH of the Tigrayan chicken ecotypes and the reference population's genome.

| Ecotypes or populations/breed | N | Number of ROH | Average number of ROH / chicken | Average length of ROH Mbps | SNP number range | Total length of ROH Mbps |
| --- | --- | --- | --- | --- | --- | --- |
| Highland | 10 | 2,410 | 241±92.4 | 0.70 | 50~65,559 | 1.80 |
| Midland | 11 | 1,686 | 171.5±94.5 | 0.69 | 50~62,349 | 1.30 |
| Lowland | 8 | 1,025 | 128.4±27.8 | 0.59 | 50~29,507 | 0.62 |
| Alfamidir | 10 | 7,565 | 756.6±93.3 | 0.27 | 102~98,208 | 2.01 |
| Gesses | 10 | 6,520 | 652.1±48.9 | 0.30 | 100~150,258 | 1.98 |
| Gijet | 9 | 4,175 | 463.9±87.3 | 0.46 | 101~162,835 | 1.96 |
| Hadush_Adi | 9 | 3,872 | 430.2±22.1 | 0.50 | 100~144,963 | 1.92 |
| Hugub | 10 | 4,905 | 470.5±36.1 | 0.45 | 100~152,673 | 2.2 |
| Jarso | 14 | 9,121 | 651.5±82.3 | 0.37 | 100~109,777 | 3.4 |
| Meseret | 10 | 4,521 | 452.1±87.1 | 0.35 | 102~136,454 | 1.7 |
| Metkilimat | 10 | 4,536 | 453.7±120.6 | 0.42 | 101~179,676 | 1.95 |
| Mihquan | 10 | 4,080 | 418±42.6 | 0.34 | 100~131,621 | 1.4 |
| Kumato | 10 | 4,042 | 404.2±167.5 | 0.38 | 101~131,196 | 1.7 |
| Negasi_Amba | 10 | 7,090 | 699.1±129.5 | 0.29 | 100~94,735 | 1.9 |
| Gallus_Fayoumi | 2 | 228 | 113.5±37.5 | 0.60 | 132~75,317 | 0.16 |
| Gallus_bankiva | 2 | 39 | 19.5±10.6 | 0.19 | 138~117,500 | 0.10 |
| Gallus_gallus | 9 | 771 | 85.7±60.4 | 0.38 | 120~112,953 | 0.17 |
| Gallus_spadiceus | 5 | 110 | 22±10.3 | 0.48 | 145~61,110 | 0.06 |
| Gallus_murghi | 3 | 482 | 161±55.5 | 0.42 | 123~72,528 | 0.18 |
| Gallus_Saudi | 5 | 270 | 54.2±55.4 | 0.18 | 114~5,249 | 0.05 |
| West_Africa | 2 | 386 | 193±62.2 | 0.29 | 112~14,881 | 0.11 |

N = number of samples; ROH = run of homozygosity; Mbps = million base-pair

Suppl. Table S8. Weighted *F*_ST_ (genetic distance) values for Tigrayan indigenous chicken ecotypes and the reference populations.

|  | EH | EM | EL | AM | NA | Ja | Ku | Gj | Mes | ML | Hu | Ge | HA | Mi | Fa | WA | Sa | GS | GM | GB | GG |
| --- | --- | --- | --- | --- | --- | --- | --- | --- | --- | --- | --- | --- | --- | --- | --- | --- | --- | --- | --- | --- | --- |
| Highland | 0 | 0.01 | 0.03 | 0.03 | 0.03 | 0.12 | 0.06 | 0.04 | 0.04 | 0.04 | 0.10 | 0.03 | 0.06 | 0.06 | 0.23 | 0.18 | 0.13 | 0.20 | 0.28 | 0.27 | 0.23 |
| Midland |  | 0 | 0.03 | 0.04 | 0.03 | 0.09 | 0.03 | 0.00 | 0.00 | 0.00 | 0.07 | 0.03 | 0.02 | 0.01 | 0.17 | 0.10 | 0.08 | 0.13 | 0.21 | 0.19 | 0.16 |
| Lowland |  |  | 0 | 0.06 | 0.06 | 0.11 | 0.05 | 0.05 | 0.05 | 0.04 | 0.10 | 0.03 | 0.06 | 0.05 | 0.19 | 0.14 | 0.09 | 0.17 | 0.25 | 0.24 | 0.20 |
| Alfamidir |  |  |  | 0 | 0.00 | 0.10 | 0.05 | 0.05 | 0.05 | 0.04 | 0.08 | 0.03 | 0.06 | 0.06 | 0.22 | 0.17 | 0.13 | 0.19 | 0.27 | 0.26 | 0.23 |
| Negasi_Amba |  |  |  |  | 0 | 0.10 | 0.04 | 0.04 | 0.04 | 0.04 | 0.08 | 0.03 | 0.06 | 0.05 | 0.21 | 0.16 | 0.12 | 0.18 | 0.25 | 0.24 | 0.22 |
| Jarso |  |  |  |  |  | 0 | 0.09 | 0.10 | 0.10 | 0.09 | 0.07 | 0.11 | 0.10 | 0.09 | 0.22 | 0.16 | 0.13 | 0.18 | 0.26 | 0.25 | 0.21 |
| Kumato |  |  |  |  |  |  | 0 | 0.03 | 0.02 | 0.02 | 0.06 | 0.04 | 0.03 | 0.03 | 0.15 | 0.08 | 0.06 | 0.12 | 0.19 | 0.17 | 0.15 |
| Gijet |  |  |  |  |  |  |  | 0 | 0.00 | 0.00 | 0.07 | 0.03 | 0.01 | 0.01 | 0.16 | 0.08 | 0.07 | 0.12 | 0.19 | 0.17 | 0.16 |
| Meseret |  |  |  |  |  |  |  |  | 0 | 0.00 | 0.07 | 0.04 | 0.02 | 0.01 | 0.16 | 0.09 | 0.07 | 0.11 | 0.19 | 0.17 | 0.15 |
| Metkilimat |  |  |  |  |  |  |  |  |  | 0 | 0.07 | 0.03 | 0.01 | 0.01 | 0.16 | 0.08 | 0.07 | 0.12 | 0.19 | 0.17 | 0.16 |
| Hugub |  |  |  |  |  |  |  |  |  |  | 0 | 0.09 | 0.08 | 0.07 | 0.20 | 0.14 | 0.11 | 0.16 | 0.24 | 0.23 | 0.20 |
| Gesses |  |  |  |  |  |  |  |  |  |  |  | 0 | 0.05 | 0.04 | 0.20 | 0.15 | 0.11 | 0.17 | 0.24 | 0.23 | 0.21 |
| Hadush_Adi |  |  |  |  |  |  |  |  |  |  |  |  | 0 | 0.00 | 0.15 | 0.08 | 0.07 | 0.12 | 0.19 | 0.17 | 0.16 |
| Mihquan |  |  |  |  |  |  |  |  |  |  |  |  |  | 0 | 0.15 | 0.08 | 0.06 | 0.11 | 0.18 | 0.17 | 0.15 |
| Gallus_Fayoumi |  |  |  |  |  |  |  |  |  |  |  |  |  |  | 0 | 0.25 | 0.14 | 0.18 | 0.34 | 0.40 | 0.23 |
| West_Africa |  |  |  |  |  |  |  |  |  |  |  |  |  |  |  | 0 | 0.08 | 0.10 | 0.24 | 0.26 | 0.15 |
| Gallus_Saudi |  |  |  |  |  |  |  |  |  |  |  |  |  |  |  |  | 0 | 0.11 | 0.23 | 0.24 | 0.15 |
| Gallus_spadiceus |  |  |  |  |  |  |  |  |  |  |  |  |  |  |  |  |  | 0 | 0.11 | 0.09 | 0.08 |
| Gallus_murghi |  |  |  |  |  |  |  |  |  |  |  |  |  |  |  |  |  |  | 0 | 0.25 | 0.20 |
| Gallus_bankiva |  |  |  |  |  |  |  |  |  |  |  |  |  |  |  |  |  |  |  | 0 | 0.17 |
| Gallus_gallus |  |  |  |  |  |  |  |  |  |  |  |  |  |  |  |  |  |  |  |  | 0 |

EH = highland; EM = midland; EL = lowland; AM = Alfamidir, NA = Negasi_Amba; Ja = Jarso; Ku = Kumato; Gi = Gijet; Mes = Meseret; ML = Metkilimat; Hu= Hugub; Ge = Gesses; HA = Hadush_Adi; Mi = Mihquan; FA = Gallus_Fayoumi; WA = West_Africa; Sa = Gallus_Saudi; GS = Gallus_spadiceus; GM = Gallus_murghi; GB = Gallus_bankiva; GG= Gallus_gallus

Suppl. Table S9. All SNP consequences across the Tigrayan indigenous chicken ecotypes genome.

| Consequence type | Ecotypes | | |
| --- | --- | --- | --- |
|  | Highland | Midland | Lowland |
| Splice donor variant | 460 | 593 | 494 |
| Splice acceptor variant | 319 | 430 | 344 |
| Stop gained | 384 | 472 | 399 |
| Stop lost | 59 | 84 | 69 |
| Start lost | 136 | 203 | 132 |
| Missense variant | 44,924 | 60,327 | 47,699 |
| Splice region variant | 25,083 | 32,480 | 25,969 |
| Synonymous variant | 112,591 | 147,227 | 117,858 |
| Stop retained variant | 49 | 68 | 46 |
| Coding sequence variant | 3 | 6 | 4 |
| Mature miRNA variant | 134 | 176 | 129 |
| 5_prime_UTR_variant | 20,460 | 27,077 | 21,231 |
| 3_prime_UTR_variant | 69,588 | 91,099 | 70,421 |
| Non-coding transcript exon variant | 84,862 | 107,131 | 88,181 |
| Intron variant | 8,391,295 | 10,583,075 | 8,709,363 |
| Non-coding transcript variant | 1,065,260 | 1,323,655 | 1,084,561 |
| Upstream gene variant | 1,338,395 | 1,734,402 | 1,397,326 |
| Downstream gene variant | 1,282,171 | 1,656,131 | 1,334,762 |
| Intergenic variant | 2,061,181 | 2,558,241 | 2,132,276 |

miRNA = micro Ribonucleic acid; UTR = untranslated regions.

Suppl. Table S10. Coding consequence of Tigrayan indigenous chicken ecotypes SNPs.

|  | Highland | Midland | Lowland |
| --- | --- | --- | --- |
| Stop gained | 384 | 472 | 399 |
| Stop lost | 59 | 84 | 69 |
| Start lost | 136 | 203 | 132 |
| Missense variant | 44,924 | 60,327 | 47,699 |
| Synonymous variant | 112,591 | 147,227 | 117,858 |
| Stop retained variant | 49 | 68 | 46 |
| Coding sequence variant | 3 | 6 | 4 |
| Total | 158146 | 208387 | 166207 |

Suppl. Table S11. Variant Effect Predictor (VEP) annotation of private SNPs across the genome of the Tigrayan indigenous chicken ecotypes.

| Consequence type | Ecotypes | | |
| --- | --- | --- | --- |
|  | Highland | Midland | Lowland |
| 3_prime_UTR_variant | 2878 | 5135 | 3467 |
| 5_prime_UTR_variant | 659 | 1169 | 803 |
| Coding sequence variant | 0 | 1 | 0 |
| Downstream gene variant | 44835 | 82096 | 55993 |
| Intergenic variant | 380438 | 670664 | 455032 |
| Intron variant | 231443 | 421359 | 290696 |
| Intron variant, non-coding transcript variant | 67899 | 121363 | 80727 |
| Mature miRNA variant | 3 | 6 | 7 |
| Missense variant (non-synonymous variants) | 1995 | 3634 | 2691 |
| Missense variant, splice region variant | 41 | 69 | 41 |
| Noncoding transcript exon variant | 4290 | 7781 | 5129 |
| Splice acceptor variant | 9 | 6 | 3 |
| Splice acceptor variant, non-coding transcript variant | 9 | 26 | 13 |
| Splice donor variant | 5 | 9 | 8 |
| Splice donor variant, non-coding transcript variant | 20 | 31 | 22 |
| Splice region variant, 3_prime_UTR_variant | 2 | 2 | 1 |
| Splice region variant, 5_prime_UTR_variant | 7 | 16 | 10 |
| Splice region variant, intron variant | 619 | 1144 | 745 |
| Splice region variant, intron variant, non-coding transcript variant | 116 | 208 | 146 |
| Splice region variant, non-coding transcript exon variant | 73 | 82 | 71 |
| Splice region variant, synonymous variant | 125 | 188 | 111 |
| Start lost | 6 | 11 | 4 |
| Stop gained | 18 | 38 | 27 |
| Stop gained, splice region variant | 1 | 1 | 0 |
| Start lost, splice region variant | 0 | 0 | 1 |
| Stop lost | 2 | 6 | 3 |
| Stop retained variant | 5 | 3 | 0 |
| Synonymous variant | 4173 | 7644 | 5121 |
| Upstream gene variant | 51027 | 90500 | 62387 |
| Total private SNPs | 790698 | 1413192 | 963259 |

miRNA = micro-Ribonucleic acid; UTR = untranslated regions; SNPs = single nucleotide polymorphism.

Suppl. Table S12. The count of deleterious SNPs in the coding and splicing regions of the Tigrayan indigenous chicken genome.

| Ecotypes | Number of deleterious missense variants | Number of deleterious missense splice region variants |
| --- | --- | --- |
| Highland | 463 | 8 |
| Midland | 808 | 15 |
| Lowland | 608 | 12 |

Suppl. Table S13. Overlapping private SNPs, candidate GO: terms, and KEGG pathways, and their respective genes common for the highland, midland and lowland Tigrayan indigenous chicken ecotypes.

| Category | GO: term and KEGG | Count | Gene list |
| --- | --- | --- | --- |
| GOTERM_MF_DIRECT | Lipid transporter activity (GO:0005319) | 4 | *VTG1, VTG2, VTG3* and *APOB* |
| GOTERM_MF_DIRECT | Iron ion binding (GO:0005506) | 16 | *ACO2, AOX1, AOX2, CYGB, CYP2C23A, CYP2C23B, CYP3A5, CYP46A1, P3H2, PAH, PRCP, TF, XDH, AOX2, CYP11A1* and *TPH1* |
| GOTERM_CC_DIRECT | Integral component of membrane (GO:0016021) | 269 | *ABCB1, ABCC1, ABCD2, ABCG4, ABHD12, ACE, ACVR2B, ADAM33, ADAM9, ADGRA1, ADORA1, ADPRH, ADRA1A, ADRA1B, ADRA2A, ADRB1, ADTRP, ALC, ALG10, ALG6, AMHR2, ANO10, AQP1, AQP2, AREG, ASPHD1, ATP12A, ATP13A4, ATP6V0A4, B3GALT5, BACE2, BDKRB1, CANX, CAV2, CCK1R, CCR2, CCR6, CCR8L, CCR9, CD44, CDH22, CDH6, CDHR1, CERS3, CGNRH-R, CHST8, CLDN2, CLPTM1L, CLSTN1, CMTM4, CNTNAP5, COL22A1, COLEC12, COX10, CPT1A, CRHR2, CRTAM, CX3CL1, CXCR5, CXCR7, CYB561, CYBRD1, CYP3A5, DD1, DDO, DEGS1, DISP3, DPP4, DRAM1, EDNRB, EGF, ELOVL3, ENTPD3, EVC, EVC2, FAM155B, FAM210B, FAM69C, FASLG, FGF6, FGFRL1, FMO3, FUT4, FZD10, FZD7, G6PC2, GALNT3, GALR1L, GASZ, GCNT2, GDPD4, GHSR, GIGYF2, GIPR, GJA5, GJB5, GJD4, GPAT3, GPR107, GPR137C, GPR15, GPR37, GPR37L1, GPR83, GPR83L, GREB1L, GXYLT1, HTR7, IFNAR1, IFNAR2, IFNGR1, IL10RA, IL1R1, IL4R, IMPG2, INTS2, JPH3, KCNA3, KCNA4, KCND2, KCNG2, KDR, KIAA1524, LAMP1, LAMP3, LARGE1, LARGE2, LAT2, LECT1, LETM1, LICOS, LINGO3, LOC101747503, LOC101747844, LOC101750836, LOC107050476, LOC112532140, LOC414835, LOC417937, LOC422926, LOC428421, LOC431250, LOC769317, LOC771308, MANSC1, MARCO, MC4R, MCAM, MET, MFN1, MFSD11, MFSD4A, MGAT4A, MGAT5, MLF2, MMR1L3, MMR1L4, MRC1, MSTN, MTR, NAT10, NCX1, NINJ2, NMUR1, NMUR2, NOC4L, NPBWR1, NPFFR2, NRXN3, NSDHL, NUS1, OCA2, OPN5L1, OPRK1, OR52B2L, PCDHGA2, PDE3B, PEMT, PGRMC2, PIGA, PIGV, PKD2, PLLP, PMP22, PRLHR, PRLHR2, PROKR1, PRTG, PTK7, PTPRU, RNF144B, ROS1, RTN1, RYR3, S1PR2, SDC1, SDK1, SERINC2, SLC15A1, SLC15A2, SLC16A1, SLC16A8, SLC17A9, SLC18A3, SLC22A18, SLC22A4, SLC25A33, SLC2A11L4, SLC2A13, SLC2A8, SLC30A4, SLC30A6, SLC30A8, SLC41A2, SLC45A4, SLC46A3, SLC47A1, SLC48A1, SLC5A8, SLC7A1, SLC7A11, SLC7A13, SLC7A7, SLC7A9, SLCO1C1, SLCO2A1, SMIM8, SMO, SSTR5, ST6GALNAC1, ST6GALNAC5, ST7L, STING1, STX11, SYBU, TAS2R40, TLR1A, TLR3, TLR4, TMEM123, TMEM125, TMEM144, TMEM17, TMEM178B, TMEM182, TMEM184B, TMEM207, TMEM209, TMEM255A, TMEM255B, TMEM59, TMEM8C, TMPRSS2, TNFRSF13C, TNFRSF1B, TNFRSF8, TNFSF10, TNMD, TRPC6, TSPAN15, UGT8L, VSIG1, WNT6, XK, XKR9, YIPF7, YME1L1, ZP2* and *ZP4,* |
| GOTERM_CC_DIRECT | Nuclear replication fork (GO:0043596) | 5 | *WDHD1, ETAA1, PARP1, RPL37A* and *MMS22L* |

Suppl. Table S14. Overlapping private SNPs, candidate GO: terms, KEGG and their respective genes common for the highland and lowland Tigrayan indigenous chicken ecotypes.

| Category | GO: term and KEGG | Count | Gene list |
| --- | --- | --- | --- |
| KEGG_PATHWAY | Fanconi anaemia pathway (gga03460) | 12 | *ATR, ATRIP, BLM, BRCA2, FANCA, FANCB, FANCD2,*  *FANCI, FANCL, MLH1, POLH* and *SLX4* |
| GOTERM_BP_DIRECT | Double-strand break repair via homologous recombination (GO:0000724) | 10 | *ATM, BLM, BRCA2, HUS1, MCM9, PRKDC, RAD54B, SMC6, MMS22L* and *SFPQ* |
| GOTERM_BP_DIRECT | Lipid transport (GO:0006869) | 5 | *VTG1, VTG2, VTG3, ABCC1* and *APOB* |
| GOTERM_MF_DIRECT | Nutrient reservoir activity (GO:0045735) | 3 | *VTG1, VTG2* and *VTG3* |
| GOTERM_BP_DIRECT | Cellular response to estrogen stimulus (GO:0071391) | 5 | *VTG1, AR, VTG2, VTG3* and *ESR2* |

Suppl. Table S15. Overlapping private SNPs, candidate GO: terms, KEGG and their respective genes common for the midland and lowland Tigrayan indigenous chicken ecotypes.

| Category | GO: term and KEGG | Count | Gene list |
| --- | --- | --- | --- |
| KEGG_PATHWAY | Homologous recombination (gga03440) | 10 | *ATM, BARD1, BLM, BRCA2, POLD3, RAD54B, RAD54L, RBBP8, TOPBP1* and *UIMC* |
| KEGG_PATHWAY | Cytokine-cytokine receptor interaction (gga04060) | 43 | *ACVR2B, AMHR2, BMP3, BMPR1B, CCL4, CCR2, CCR4, CCR6, CCR8L, CHALK5, CSF1R, CX3CL1, CXCR5, CXCR7, EDA2R, FASLG, GDF6, GDF7, IFNAR1, IFNAR2, IFNGR1, IL10, IL10RA, IL17A, IL18R1, IL1R1, IL1R2, IL1RL2, IL20RB, IL22, IL25, IL2RA, IL4R, LEPR, LOC107049250, MSTN, RELT, TGFB3, TNFRSF13C, TNFRSF1B, TNFRSF8, TNFSF10* and *TNFSF11* |
| GOTERM_MF_DIRECT | Aspartic-type endopeptidase activity (GO:0004190) | 7 | *BACE2, CASP7, CPGC, CTSEAL, DDI2, LOC107050476* and *LOC112532140* |
| GOTERM_MF_DIRECT | G-protein coupled receptor activity (GO:0004930) | 28 | *ADGRA1, ADRA1A, ADRA1B, BDKRB1, CCK1R, CRHR2, CXCR7, FZD10, FZD7, GALR1L, GIPR, GPR174, GPR19, GPR37, GPR83, HTR7, LOC101747503, LOC101750836, LOC431250, LOC769317, MTR, NMUR1, OPN5L1, OPRK1, OR52B2L, SMO, SS3R* and *TAS2R40* |
| GOTERM_CC_DIRECT | Membrane (GO:0016020) | 52 | *ABCC1, ABHD12, ACE, ACVR2B, CHALK5, CNTNAP5, COL4A1, COLEC12, CYP3A5, DISP3, ENTPD3, ENTPD5, FZD10, FZD7, GIGYF2, HHIPL1, IMPG2, INSR, KCNG2, KIAA1524, LAMP1, LECT1, LEPR, LIMK1, LY6E, MGAT3, NCX1, NRXN3, PALMD, PDGFB, PGRMC2, PIK3C2A, PIK3C2B, PIT54, PNPLA2, PON1, PRKD3, RASL10A, SLC45A4, SMO, SNTB1, SPRY2, ST7L, STK10, TAS2R40, TFCP2L1, TIAM1, TIAM2, TMEM178B, TMEM209, TNFSF10 and TNFSF11* |
| GOTERM_MF_DIRECT | Carbohydrate binding (GO:0030246) | 13 | *LOC428421, ACAN, CMBL, CNTN2, COLEC12, GALNT3, LOC428421, MASP1, MBL, MMR1L3, MMR1L4, MRC1* and *SI* |

Suppl. Table S16. Overlapping private SNPs, candidate GO terms, and KEGG pathways, and their respective genes common for the highland indigenous Tigrayan chicken ecotypes.

| Category | GO term and KEGG | Count | *P*-value | Gene list |
| --- | --- | --- | --- | --- |
| KEGG_PATHWAY | Peroxisome (gga04146) | 12 | *0.004* | *ABCD2, SLC25A17, ACOX2, PEX6, EPHX2, ACSL6, HACL1, HAO1, PEX1, ACSL3, XDH* and *DDO* |
| KEGG_PATHWAY | ECM-receptor interaction (gga04512) | 11 | *0.0176* | *FRAS1, RELN, LAMB3, LAMA1, LAMA4, ITGB3, COL4A4, LAMA3, HMMR, FREM2* and *ITGA9* |
| GOTERM_CC_DIRECT | Axoneme (GO:0005930) | 4 | *0.0246* | *ARL13B, LCA5, BBS7 and DRC1* |
| GOTERM_BP_DIRECT | DNA repair (GO:0006281) | 8 | *0.0437* | *FANCI, WDHD1, PARP1, FANCL, FANCD2, ACTR5, RPL37A* and *ATM* |
| GOTERM_BP_DIRECT | Enteric nervous system development (GO:0048484) | 3 | *0.04* | *EDNRB, SOX8* and *SOX10* |

Suppl. Table S17. Overlapping private SNPs, candidate GO terms, and KEGG pathways, and their respective genes common for the midland indigenous Tigrayan chicken ecotypes.

| Category | GO term and KEGG | Count | *P*-value | Gene list |
| --- | --- | --- | --- | --- |
| KEGG_PATHWAY | One carbon pool by folate (gga00670) | 6 | 0.013 | *GART-B, MTHFD1, MTHFD1L, MTHFD2L, MTR* and *MTHFS* |
| KEGG_PATHWAY | ABC transporters (gga02010) | 10 | 0.008 | *ABCC2, ABCB1, ABCA5, ABCB1LA, ABCA3, ABCB9, ABCA8, ABCA12, ABCG4* and *ABCC13* |
| KEGG_PATHWAY | Neuroactive ligand-receptor interaction (gga04080) | 41 | 0.043 | *CHRM2, NPFFR2, LOC422147, GALR1L, GIPR, MTR, GPR83, ADRA1B, RXFP1, ADRA1A, CRHR2, CYSLTR1, GLRA2, EDNRB, HTR7, CYSLTR2, PGRMC2, GRM8, HRH4, ADORA1, LOC100859173, BDKRB1, PRLHR, UTS2R, NMB, CCK1R, EDN2, GABRA3, HTR1D, TAAR5, OPRK1, UTS2R2, GABRG1, MC3R, PRLHR2, PNOC, NMUR1, CGNRH-R* and *F2RL3* |
| KEGG_PATHWAY | Hedgehog signalling pathway (gga04340) | 12 | 0.002 | *EVC2, HHATL, SHH, HHAT, GPR161, SUFU, MYO18B, BOC, LRP2, GLI1, DISP1* and *CDON* |
| GOTERM_BP_DIRECT | Cell fate specification (GO:0001708) | 5 | 0.035 | *EOMES, TBX15, SHH, TBX22* and *TBXT* |
| GOTERM_MF_DIRECT | NAD+ nucleosidase activity (GO:0003953) | 5 | 0.038 | *BST1, IL1R1, TLR1A, TLR4* and *TLR3* |
| GOTERM_MF_DIRECT | Transmembrane receptor protein tyrosine kinase activity (GO:0004714) | 7 | 0.031 | *EPHB6, FLT1, INSR, KDR, ROS1, EPHB1* and *EPHB3* |
| GOTERM_MF_DIRECT | Neuropeptide Y receptor activity (GO:0004983) | 4 | 0.020 | *PROKR1, PRLHR2, PRLHR* and *GPR83* |
| GOTERM_MF_DIRECT | Scavenger receptor activity (GO:0005044) | 5 | 0.017 | *COL22A1, NTL, TMPRSS2, HHIPL1* and *CXCR7* |
| GOTERM_CC_DIRECT | Integral component of plasma membrane (GO:0005887) | 44 | 0.0002 | *EPHB6, CHRM2, FLT1, GALR1L, LOC768760, PCDH10, MTR, ADRA1B, APCDD1, RXFP1, ADRA1A, EDA2R, CYSLTR1, HTR7, CYSLTR2, GJA3, GJA5, KDR, SLC16A8, EPHB1, ROS1, SLC16A3, EPHB3, UTS2R, SLC15A1, CCK1R, SLC16A1, DSCAM, RFC1, INSR, HTR1D, OPRK1, NCX1, UTS2R2, TMEM130, GABRG1, SLCO1C1, SLCO3A1, PRPH2, RHAG, FAM26F, TLR4* and *TLR3* |
| GOTERM_BP_DIRECT | Smooth muscle contraction (GO:0006939) | 3 | 0.026 | *HTR7, HTR1D* and *NMUR1* |
| GOTERM_BP_DIRECT | Homophilic cell adhesion via plasma membrane adhesion molecules (GO:0007156) | 12 | 0.003 | *PRTG, CDH6, SDK1, DSCAM, LOC768760, LOC414835, PCDH10, PCDHGA2, MCAM, CDHR1, CLSTN1* and *CDH22* |
| GOTERM_BP_DIRECT | Cell surface receptor signalling pathway (GO:0007166) | 6 | 0.028 | *CYSLTR1, ADGRA1, GIPR, RANBP10, CBL* and *CRHR2* |
| GOTERM_BP_DIRECT | Cell-cell signalling (GO:0007267) | 8 | 0.006 | *GJD4, SHH, GJA3, GJA5, GJB5, ADRA1B, FJX1 and ADRA1A* |
| GOTERM_BP_DIRECT | Mitochondrial fusion (GO:0008053) | 3 | 0.039 | *MFN1, BAK1* and *GDAP* |
| GOTERM_MF_DIRECT | G-protein coupled peptide receptor activity (GO:0008528) | 7 | 0.013 | *UTS2R, CYSLTR1, CYSLTR2, GALR1L, GPR37L1, UTS2R2* and *RXFP1* |
| GOTERM_MF_DIRECT | Lactate transmembrane transporter activity (GO:0015129) | 4 | 0.006 | *SLC5A8, SLC16A1, SLC16A8* and *SLC16A3* |
| GOTERM_MF_DIRECT | L-amino acid transmembrane transporter activity (GO:0015179) | 4 | 0.006 | *SLC7A7, SLC7A9, SLC7A13* and *SLC7A11* |
| GOTERM_MF_DIRECT | Symporter activity (GO:0015293) | 5 | 0.045 | *SLC15A1, SLC5A8, SLC15A2, SLC16A8* and *SLC16A3* |
| GOTERM_BP_DIRECT | Positive regulation of interferon-alpha production (GO:0032727) | 4 | 0.026 | *IFIH1, DHX36, TLR4* and *TLR3* |
| GOTERM_BP_DIRECT | Positive regulation of interferon-beta production (GO:0032728) | 4 | 0.042 | *IFIH1, STING1, TLR4* and *TLR3* |
| GOTERM_BP_DIRECT | Positive regulation of kinase activity (GO:0033674) | 7 | 0.018 | *EPHB6, FLT1, INSR, KDR, ROS1, EPHB1* and *EPHB3* |
| GOTERM_BP_DIRECT | Vasoconstriction (GO:0042310) | 3 | 0.042 | *EDNRB, HTR7* amd *HTR1D* |
| GOTERM_CC_DIRECT | Synapse (GO:0045202) | 18 | 0.041 | *CHRM2, CYFIP2, ACHE, SLC16A1, DSCAM, CLSTN1, CHAT, HTR1D, DYTN, GABRG1, TIAM2, SDK1, TIAM1, HTR7, GPC1, PNOC* and *ADORA1* |

Suppl. Table S18. Overlapping private SNPs, candidate GO terms, and KEGG pathways, and their respective genes common for the lowland indigenous Tigrayan chicken ecotypes.

| Category | GO term and KEGG | Count | *P*-value | Gene list |
| --- | --- | --- | --- | --- |
| GOTERM_MF_DIRECT | Calcium-dependent cysteine-type endopeptidase activity (GO:0004198) | 3 | 0.047 | *CAPN8, CAPN2* and *CAPN1* |
| GOTERM_MF_DIRECT | ATP binding (GO:0005524) | 44 | 0.049 | *MYLK2, BLM, CIITA, FLT1, PRKDC, MCM9, FLT4, NAT10, KIF11, HSPA13, RAD54B, GK5, IFIH1, STK10, KDR, PIP4K2A, NEK2, PIP4K2B, ASCC3, BUB1, SCYL2, TRAP1, RRM1, ABCC1, EPHA7, RFC1, ATAD5, ENTPD3, LIMK1, ABCA4, DDX10, MASTL, MLH1, ACVR2B, CPS1, PTK7, ATP13A4, ATM, BMPR1B, ABCG4, MET, GALK2* and *MCM2* |
| GOTERM_BP_DIRECT | Protein glycosylation (GO:0006486) | 7 | 0.046 | *NUS1, ST6GALNAC1, GCNT2, B3GALT5, LARGE1, ST6GALNAC5* and *FUT4* |
| GOTERM_BP_DIRECT | Proteolysis (GO:0006508) | 9 | 0.004 | *CAPN8, PREPL, CASP7, RELN, FAP, CAPN2, UFSP2, CNDP1 and TGM2* |
| GOTERM_BP_DIRECT | G-protein coupled receptor signalling pathway (GO:0007186) | 12 | 0.026 | *TAS2R40, RAMP3, CCK1R, CCLI8, SMO, FZD7, BDKRB1, NMUR1, MTR, CX3CL1, PIK3R5* and *GPR19* |
| GOTERM_BP_DIRECT | Cardiac muscle hypertrophy in response to stress (GO:0014898) | 3 | 0.021 | *KDM4A, GATA6* and *KLF15* |
| GOTERM_CC_DIRECT | Cell junction (GO:0030054) | 8 | 0.006 | *TSPAN15, PTPRU, FAP, TP53BP2, KDR, XIRP1, TP73* and *FOXA2* |
| GOTERM_BP_DIRECT | Response to estradiol (GO:0032355) | 4 | 0.014 | *FOXA1, VTG1, VTG2* and *VTG3* |
| GOTERM_MF_DIRECT | Histone demethylase activity (GO:0032452) | 3 | 0.047 | *KDM5A, KDM4A* and *JARID2* |
| GOTERM_MF_DIRECT | Peptide binding (GO:0042277) | 5 | 0.048 | *SS3R, BDKRB1, MTR, GPR37L1* and *GPR19* |
| GOTERM_BP_DIRECT | Cholesterol homeostasis (GO:0042632) | 6 | 0.016 | *NUS1, DISP3, SEC24A, APOB, ABCG4* and *MUC6* |
| GOTERM_CC_DIRECT | Intracellular membrane-bounded organelle (GO:0043231) | 9 | 0.011 | *BARD1, PHLPP1, BDH1A, UGT8L, ANO10, CYP2C23A, FMO3, RAB11FIP2* and *MUC6* |
| GOTERM_BP_DIRECT | Protein homooligomerization (GO:0051260) | 7 | 0.040 | *LETM1, KCNG2, BLM, KCND2, KCNA3, KCTD12* and *TGM2* |
| GOTERM_MF_DIRECT | FAD binding (GO:0071949) | 3 | 0.046 | *AOX2, AOX1* and *PRODH* |
